# Longitudinal associations between domains of flourishing

**DOI:** 10.1038/s41598-022-06626-5

**Published:** 2022-02-17

**Authors:** Ying Chen, Dorota Weziak-Bialowolska, Matthew T. Lee, Piotr Bialowolski, Eileen McNeely, Tyler J. VanderWeele

**Affiliations:** 1 Human Flourishing Program, Harvard Institute for Quantitative Social Science, Cambridge, MA USA; 2 Department of Epidemiology, Harvard T. H. Chan School of Public Health, Boston, MA USA; 3 Sustainability and Health Initiative (SHINE), Department of Environmental Health, Harvard T.H. Chan School of Public Health, Boston, MA USA

**Keywords:** Epidemiology, Human behaviour

## Abstract

The longitudinal interrelationships between domains of human well-being or flourishing remain understudied empirically. While different aspects of flourishing may be sought as their own end, it is also the case that well-being in one domain may influence well-being in other domains. Using longitudinal data form a sample of employees from a large national employer in the United States (N = 1209, mean age = 43.52 years, age range 20–74 years), this study examined the temporal associations between various domains of flourishing, based on a 40-item index that assessed six domains of flourishing. These domains include emotional health, physical health, meaning and purpose, character strengths, social connectedness, and financial security. A set of linear regression models were used to regress subsequent composite flourishing on flourishing domain-specific scores at baseline. The results indicated that all domains were each independently associated with greater composite flourishing subsequently. The strongest and most robust links were observed for meaning and purpose (β = 0.19, 95% confidence interval [CI] 0.13, 0.25), social connectedness (β = 0.17, 95% CI 0.12, 0.22), and financial security (β = 0.32, 95% CI 0.28, 0.37). Further analyses that regressed subsequent composite flourishing on *individual* item indicators at baseline suggested that, out of all 40 items, one item under the character domain “I always act to promote good in all circumstances, even in difficult and challenging situations” and one item in the physical health domain (“Based on my past health, I expect to be healthy long into the future”) had the most robust association with subsequent composite flourishing. Implications of these results for understanding the constituents of a flourishing life and for refinement of the flourishing assessments are discussed.

## Introduction

The concept of “flourishing” might be understood as “a state in which all aspects of a person’s life are good”^1^. While there have been discussions regarding the constituents of a flourishing life, there is general consensus that complete well-being or flourishing is a complex and multi-faceted concept^2^. As such, a number of composite measures of flourishing were proposed, which, however, have focused primarily on assessing psychological wellbeing^2–5^. Such measures of psychological flourishing may, nevertheless, fail to assess some important aspects of life other than psychological wellbeing that are arguably also essential to holistic wellbeing. Specifically, some long-standing traditions suggest that flourishing is a broad notion that consists of domains beyond one’s mental state and feelings towards life^1^. For instance, virtue has often been considered in the philosophical literature as central to a flourishing life, and Aristotle argued centuries ago that “happiness is attained by actions in accordance with virtue”^6,7^. Likewise, physical health is often considered as an outcome in studies of psychological wellbeing, and thus not included in measures of psychological flourishing. However, if the principal interest is to assess complete wellbeing, one may argue that physical health is essential to individuals’ sense of wholeness and should arguably also be included in assessments of flourishing^1^.

To facilitate further discussions on flourishing as broadly conceived, VanderWeele proposed a conceptual model of human flourishing^1^ considering multiple domains that are often conceived as essential to a flourishing life in philosophical and social science traditions^8,9^. These include (1) happiness and life satisfaction; (2) physical and mental health; (3) meaning and purpose; (4) character and virtue; and (5) close social relationships. Each of these domains arguably satisfies two criteria including: (1) it is nearly universally desired, and (2) it is generally considered as an end in itself. While financial and material stability are generally not viewed as ends for pursuit in themselves, sufficient resource stability can be an important precondition for sustaining well-being in other domains. Therefore, VanderWeele also suggested an additional “enabling” domain of (6) financial and material stability which may be necessary for “preserving those goods that are their own ends”^1^, when considering sustained flourishing over time. While there have been prior measures for each of these domains, there is not a measure that assesses the broader components of composite flourishing. To this end, VanderWeele and colleagues proposed a 40-item flourishing index^10^ that mostly consists of selected questionnaire items from previously validated measures for each of these domains. Because individuals may view the importance of each domain differently and there is not a clear way of weighting across domains, VanderWeele and colleagues proposed to derive a summary index for composite flourishing by applying equal weights to each domain^1,10^. This proposed index has been empirically validated in a prior study^11^. It demonstrated evidence of goodness of fit in terms of traditional fit indices (i.e., CFI, TLI and RMSEA), satisfactory reliability, high test–retest correlation, adequate convergent/discriminant validity, as well as acceptable measurement invariance. The comprehensive psychometric analyses provided support that the index can be used to assess composite flourishing as well as its six domains^11^. As VanderWeele acknowledged, however, further evidence would be needed to understand the relationships between these proposed domains and to assess utility of this proposed index^1^.

Conceptually, whereas these proposed domains of flourishing are each a distinct end for pursuit in themselves, achievements or failures to live well in one domain may influence other domains^1^. There are multiple ways in which the various domains of flourishing may be related. First, there may be causal developmental relationships between the domains, which suggests that flourishing in one domain may be essential for the development of other domains^12,13^. For example, the possession of some character strengths such as honesty and justice is sometimes considered as preconditions for developing the best forms of social relationships^12^. Next, the domains of flourishing may be related to each other expressively such that the status in one domain may activate or inhibit the expression of certain dispositions in other domains^12^. For example, severe mental distress may prevent an individual from expressing his or her otherwise compassionate disposition to other people^14^. Moreover, there may be constitutive relationships between the domains of flourishing such that the possession of certain attributes in one domain may be essential for another domain to be “going well” in its fullest sense^12^. For example, an individual may not be considered as truly doing well in the domain of meaning and purpose, if the person’s purposes in life are immoral.

Empirically, there has been considerable evidence suggesting reciprocal associations between some of these proposed flourishing domains. For instance, some prior longitudinal studies suggested bi-directional associations of emotional health with physical health^15–22^, purpose in life^21–23^ and social integration^21,22^. Likewise, there has been evidence suggesting that physical health may shape subsequent emotional health^24^, sense of purpose^25,26^, social connectedness^27^ and financial status^28^, and vice versa^29–32^. In addition, increasing evidence has suggested that certain traits of character strengths were associated with subjective well-being^33,34^ and purpose in life^23,35^, though the associations of character with other domains remain less often studied. These prior studies have, however, often examined these domains in separate studies. Examining the dynamics between these domains simultaneously within an overarching framework of composite flourishing has the potential to help foster better understanding of the roles of individual domains in shaping overall flourishing and well-being across other domains. Such evidence would be important for informing discussions on theoretical models of composite flourishing, refinement of flourishing assessments, and informing public health recommendations for promoting holistic wellbeing at the population level.

To address this knowledge gap, this study examined the longitudinal associations between various domains of flourishing based on VanderWeele’s model of human flourishing, using the previously proposed set of 40 well-being questions across 6 domains^1,10,11^. The use of these numerous well-being questions allows for identification of specific aspects of well-being and potential items whose responses are most strongly related to subsequent improvements in other aspects of well-being. We hypothesized that each domain is positively associated with subsequent composite flourishing and with subsequent well-being in other domains.

## Methods

### Study participants

Participants of this study were a sample of employees from a large national self-insured services organization in the United States. The initial invitation to participate was sent in June 2018 to a random sample of 15,000 employees via the work email system. A total of 2364 participants returned the completed questionnaires, with 52 of them randomly selected to win a cash prize (ranging from $100 to $1000) as an incentive for participation. The respondents who remained employed in this institution were followed-up again in July 2019, and 1411 of them responded. Compared to participants who dropped out at follow-up, those who remained in the cohort were older, a higher percentage of female, non-Hispanic White and married, and more likely to own a house, but overall there was little difference in other baseline characteristics between these two groups (Supplementary Table S1). The present study included the participants who returned the questionnaires at both waves of the data collection and had complete data on the flourishing outcomes at the follow-up wave (N = 1209). This study was approved by the Institutional Review Board at Harvard T. H. Chan School of Public Health. All methods were carried out in accordance with relevant guidelines and regulations. Written Informed consent was obtained from all participants.

### Assessment of independent variables

#### Domains of flourishing

A previously-validated 40-item flourishing index^10,11^, developed based on VanderWeele’s theoretical model of human flourishing^1^ but adapted to the particular context and aims of the employer, was used to assess well-being in 6 domains at study baseline. These domains include (1) emotional health (e.g., “how satisfied are you with life as a whole these days”); (2) physical health (e.g., “How would you rate your physical health”); (3) meaning and purpose (e.g., “I have values and beliefs that help me understand who I am”); (4) Character strengths (e.g., “I always act to promote good in all circumstances, even in difficult and challenging situations”); (5) social connectedness (e.g., “My relationships are as satisfying as I would want them to be”); and (6) financial security (e.g., “I am able to meet my normal monthly living expenses without any difficulty”) (A full list of the items was given in Table 3 and Supplementary Table S2). The responses were measured on a 11-point Likert scale from 0 to 10. Some items were reverse coded as necessary, so that a higher score indicates greater well-being. A domain-specific score was calculated for each domain by averaging the responses across all items under the domain. This index has been empirically validated in a prior study using the same sample as the present study, and showed evidence of good fitting and satisfactory reliability, test–retest correlation and convergent/discriminant validity^11^. Cronbach’s alpha of the scales measuring each domain were reported in Supplementary Table S3.

### Assessment of dependent variables

#### Domains of flourishing

At the follow-up wave, well-being in each of the 6 domains of flourishing were again assessed with the 40-item flourishing index^10,11^. Following the same approach as the assessment of independent variables, a domain-specific score for each of the domains were calculated. Cronbach’s alpha of the scales for each domain at follow-up were shown in Supplementary Table S3.

#### Composite flourishing

An overall score for composite flourishing was calculated by averaging the domain-specific scores across all 6 domains with equal weighting^1,10^. A higher score indicates a greater extent of overall flourishing. To reduce the concern that the effects on composite flourishing may be primarily driven by autocorrelations in the same domains across time, we also calculated an alternative composite flourishing score that excluded the specific domain used as the independent variable in each analysis.

### Covariate assessment

VanderWeele’s model^1^ suggested several major pathways to flourishing including family, work, education, and religious service attendance. Each of these pathways is associated with the various proposed domains of flourishing, and may confound the temporal associations between these domains of flourishing. We, therefore, adjusted for a number of indicators for family relationships, work characteristics, socioeconomic factors and social participation in addition to other covariates, as described below.

#### Demographic and socioeconomic characteristics

Demographic covariates include age categories (≤ 30 years, 31–40 years, 41–50 years, and 50 + years), gender (female, male), race/ethnicity (non-Hispanic white, black/African American, others), and marital status (single, married, divorced, widowed, separated, non-married partner). We also considered several indicators of socioeconomic status including educational attainment (high school, some college, associated degree, bachelor degree, graduate degree), house ownership (yes, no), and the mid-point salary bands (data obtained from the human resource department of the employer).

#### Work characteristics and family caregiving responsibilities

Some prior work has suggested that job characteristics^36–38^ and family caregiving responsibilities^39,40^ may both be related to multiple aspects of health and wellbeing. Therefore, we adjusted for participants’ work hours (< 8 h/day, 8, 9–10, 11–12, 13–14, > 14 h/day), work type (exempt, non-exempt), the number of children under the age of 18 years (0, 1, 2, 3, 4, 5 +), and caregiving responsibilities for older persons at home (yes, no).

#### Social participation and civic engagement

Prior work suggested that participation in social groups and civic engagement are both associated with multiple aspects of health and wellbeing^41–44^. Therefore, we adjusted for whether the participants voted in the last presidential election (yes, no, not a registered voter), and also controlled for several indicators of social participation including frequency of religious service attendance (never, once every few months or once a year, 1–3 times a month, once a week, daily or more than once a week), spiritual practice (never, once every few months or once a year, 1–3 times a month, once a week, not daily but more than once a week, daily), participation in community groups (never, once every few months or once a year, 1–3 times a month, once a week, daily or more than once a week), and volunteering (never, once every few months or once a year, 1–3 times a month, once a week, daily or more than once a week). Some prior work suggested that subjective and objective measures of social relationships may differentially predict subsequent health and wellbeing^45,46^. The domain of social connectedness in the flourishing index assessed self-perceived social relationships, whereas the above-mentioned covariates on frequency of social participation measure objective features of the social networks.

#### Number of diagnosed physical health problems

The questionnaire items under the domain of physical health in the flourishing index assessed self-rated health. Because prior evidence has suggested that self-rated health and objective measures of health (e.g., physician-rated health) are correlated yet distinct constructs that predict subsequent health and wellbeing independently from each other^47,48^, we adjusted for the total number of physician-diagnosed major physical health problems with data obtained from medical claims as a covariate.

### Statistical analyses

All statistical analyses were performed in SAS 9.4 (SAS Institute Inc). Tests of statistical significance were two-sided.

#### Primary analyses

A set of separate linear regression models were first used to regress each of the 6 domain-specific flourishing scores at the follow-up wave (one at a time), on all of the domain-specific flourishing scores at baseline simultaneously, adjusting for covariates. Next, the primary model was reanalyzed with the composite flourishing score at follow-up as the dependent variable; and then was re-analyzed with an alternative composite flourishing score that excludes one specific domain at a time from the calculation of the composite score as the dependent variable. For easier interpretation, the dependent and the independent variables were both standardized (mean = 0, standard deviation = 1) in all models.

#### Secondary analyses

A number of secondary analyses were performed. First, the primary sets of models were reanalyzed with individual survey items of flourishing at baseline as the independent variables. Specifically, separate linear regression models were first used to regress the composite flourishing score at follow-up on individual items under each domain at baseline, adjusting for the other 5 domain-specific scores that were not taken as the independent variable, in addition to other covariates. In these analyses, the individual item indicators under one domain were first entered in the models one at a time and then included simultaneously as the independent variables. These sets of models were then reanalyzed with the alternative composite flourishing score (that excluded the domain taken as the independent variable) and with each of the 6 domain-specific scores at follow-up as the dependent variables, respectively. Next, the primary sets of models were reanalyzed with all the 40 individual survey items of flourishing at the follow-up as the dependent variables in separate models. Specifically, separate linear regression models were used to regress individual item indicators of flourishing at follow-up on all the 6 domain-specific scores of flourishing at baseline simultaneously, adjusting for covariates. These models were then reanalyzed by replacing the baseline domain scores from which the dependent variable indicator was taken, with all individual baseline item indicators in that domain.

#### Correction for multiple testing

Bonferroni correction was performed to account for multiple testing. However, Bonferroni correction is a conservative method and often produces overly conservative inference when the outcomes are correlated^49^. The practices for multiple testing vary widely in the field and this is an evolving research area^50^. Therefore, we did not use Bonferroni correction as the main lens through which we interpreted the results, but we acknowledged the different types of cutoffs that could be used in interpreting results and marked multiple p-value cutoffs both with and without Bonferroni correction in our result tables.

#### Missing data

In our full analytic sample (N = 1209), 158 had missing data on at least one flourishing survey item at baseline, and another 37 participants had missing data on covariates. We performed multiple imputation by chained equations (with 5 imputed datasets) to impute missing data on the independent variables and covariates^51–53^. As a sensitivity analysis, we also reanalyzed the primary sets of models with complete-case analyses.

## Results

### Participant characteristics

At study baseline the participants had a mean age of 43.52 years (SD = 10.43). A higher percentage of the participants were female, non-Hispanic white, and married. Over half of the participants had a bachelor’s degree or higher, owned a house, worked for 8 h per day, did not have caregiving responsibilities at home, and mostly reported a modest level of social participation (Table 1). Correlations between all study variables were reported in Supplementary Information 2.

**Table 1:** Participant characteristics at the study baseline (N = 1209).

Participant characteristics	Mean (SD) or %
**Age categories, %**	
30 years or below	11.83
31–40 years	29.94
41–50 years	28.95
50+ years	29.28
**Gender, %**	
Female	84.45
Male	15.55
**Race/Ethnicity, %**	
Non-Hispanic White	74.28
Black/African American	12.16
Others	13.56
**Marital status, %**	
Single	16.20
Married	62.47
Divorced	10.08
Widowed	1.34
Separated	1.26
Non-married partner	8.65
**Educational attainment, %**	
High school diploma or equivalent	7.78
Some college but not degree	22.58
Associate degree	13.96
Bachelor degree	34.95
Graduate degree	20.74
House ownership, %	72.36
Mid-point salary bands ($), mean (SD)^δ^	73,117.27 (342,58.59)
**Work hours (hours/day), %**	
< 8	1.17
8	51.17
9–10	35.37
11–12	6.27
13–14	0.67
> 14	5.35
**Work type, %**	
Exempt	63.61
Non-exempt	36.39
**Number of children under the age of 18 years**	
0	51.89
1	21.21
2	17.94
3	6.45
4	1.76
5+	0.75
Taking care of older persons, %	27.17
Number of health conditions, mean (SD)^±^	1.88 (2.28)
**Frequency of religious service attendance, %**	
Daily or more than once a week	5.85
Once a week	14.63
1–3 times a month	11.62
Once every few months or once a year	39.80
Never	28.09
**Frequency of spiritual practice, %**	
Daily	28.15
Not daily but more than once a week	24.73
Once a week	8.27
1–3 times a month	12.78
Once every few months or once a year	17.88
Never	8.19
**Frequency of participation in community groups, %**	
Daily or more than once a week	10.04
Once a week	8.45
1–3 times a month	15.73
Once every few months or once a year	33.89
Never	31.88
**Frequency of volunteering, %**	
Daily or more than once a week	5.03
Once a week	4.69
1–3 times a month	12.90
Once every few months or once a year	50.50
Never	26.88
**Voted in the last presidential election, %**	
Yes	82.21
No	14.09
Not a registered voter	3.69

^δ^The mid-point salary bands in this sample ranged from $33,787.81 to 246,979.16.

^±^Data on the number of health conditions were obtained from medical claims, ranging from 0 to 12.

The participants generally reported a moderate level of flourishing. The distribution of the composite flourishing and flourishing domain scores in this sample were comparable to those in a national and more representative sample^54^. Specifically, the mean score of composite flourishing was 7.48 (SD = 1.29) at baseline and 7.70 (SD = 1.34) at follow-up (range 0–10). Among all domains, meaning and purpose had the highest domain-specific score at baseline and character strengths had the highest score at follow-up. At both waves, financial security had the lowest domain-specific scores (Supplementary Table S4).

### Longitudinal associations between domains of flourishing

Composite flourishing at baseline and at follow-up were strongly associated (β = 0.59, 95% confidence interval [CI] 0.56, 0.63). All domains at baseline were positively associated with the composite flourishing score at subsequent wave, though the evidence was weak for character strengths (Table 2, Supplementary Figure S1). The associations were strongest for physical health (β = 0.17, 95% CI 0.13, 0.22), meaning and purpose (β = 0.19, 95% CI 0.13, 0.25), social connectedness (β = 0.17, 95% CI 0.12, 0.22), and for financial security (β = 0.32, 95% CI 0.28, 0.37), though, as noted above, financial security was also the domain that was most strongly associated with itself over time.

**Table 2:** Longitudinal associations between domains of flourishing (N = 1209^δ^).

Flourishing domains in 2018	Flourishing in 2019							
	Emotional health	Physical health	Meaning and purpose	Character strengths	Social connectedness	Financial security	Composite flourishing	Composite flourishing (other domains only) ±
Emotional health	0.51*** **(0.44, 0.57)**	0.08* **(0.02, 0.14)**	− 0.01 (− 0.08, 0.06)	0.01 (− 0.06, 0.08)	0.07* **(0.01, 0.14)**	0.00 (− 0.05, 0.05)	0.13*** **(0.07, 0.19)**	0.04 (− 0.02, 0.09)
Physical health	0.06* **(0.01, 0.11)**	0.65*** **(0.60, 0.70)**	0.06* **(0.01, 0.11)**	0.08** **(0.03, 0.14)**	0.03 (− 0.02, 0.08)	− 0.01 (− 0.05, 0.03)	0.17*** **(0.13, 0.22)**	0.04 (0.00, 0.09)
Meaning and purpose	0.10** **(0.03, 0.17)**	0.02 (− 0.04, 0.09)	0.60*** **(0.53, 0.67)**	0.14*** **(0.07, 0.22)**	0.12*** **(0.05, 0.19)**	0.01 (− 0.04, 0.07)	0.19*** **(0.13, 0.25)**	0.09** **(0.03, 0.15)**
Character strengths	− 0.01 (− 0.07, 0.04)	− 0.04 (− 0.10, 0.01)	− 0.01 (− 0.07, 0.04)	0.53*** **(0.47, 0.58)**	− 0.03 (− 0.08, 0.03)	− 0.03 (− 0.07, 0.02)	0.05 (0.00, 0.10)	− 0.03 (− 0.08, 0.01)
Social connectedness	0.07* **(0.01, 0.13)**	0.03 (− 0.03, 0.09)	0.09** **(0.03, 0.16)**	− 0.01 (− 0.08, 0.05)	0.55*** **(0.48, 0.61)**	0.06* **(0.01, 0.10)**	0.17*** **(0.12, 0.22)**	0.06* **(0.01, 0.12)**
Financial security	0.08** **(0.03, 0.13)**	0.08** **(0.03, 0.13)**	0.04 (− 0.01, 0.09)	0.05 (0.00, 0.11)	0.06* **(0.01, 0.11)**	0.78*** **(0.74, 0.82)**	0.32*** **(0.28, 0.37)**	0.08** **(0.03, 0.12)**

A set of separate linear regression models were used to regress each of the outcome variables at the follow-up wave (one at a time), on all of the domain-specific flourishing scores at baseline simultaneously. All models adjusted for gender, age, race, marital status, voting, education, number of children, care for older persons, home ownership, religious service attendance, spirituality, community participation, volunteering, work hours, salary bands, work type and the number of physician-diagnosed physical health problems. The domain of physical health in the flourishing index assessed self-rated health, whereas the covariate of physician-diagnosed illness was an objective indicator of health. Likewise, the domain of social connectedness in the flourishing index assessed perceived social relationships, whereas the covariates of social participation measured objective features of the social networks.

Bolded values indicate: *p < .05 before Bonferroni correction, **p < *.*01 before Bonferroni correction, ***p < *.*05 after Bonferroni correction (the p value cutoff for Bonferroni correction is p = 0.05/48 tests = 0.001).

^δ^Participants with missing data on the  dependent variables were excluded from the analyses. Multiple imputation was performed to impute missing data on the independent variables and covariates.

^±^An alternative composite flourishing score excluding the specific domain used as the independent variable in each model.

When the alternative composite flourishing score was considered (which excluded the domain used as the independent variable), the effect sizes of all domains became smaller but associations with the domains of purpose (β = 0.09, 95% CI 0.03, 0.15), social connectedness (β = 0.06, 95% CI 0.01, 0.12) and financial security (β = 0.08, 95% CI 0.03, 0.12) (Table 2, Supplementary Figure S2) remained robust. The sensitivity analysis that reanalyzed the primary models with complete-case analyses yielded similar results (Supplementary Table S5).

When the specific domains were considered as the dependent variables, participants who reported higher scores on one domain were more likely to report a high level of well-being on the same domain subsequently (Table 2, Supplementary Figures S3, S4, S5, S6, S7 and S8). Such associations held for all domains and were most pronounced in the domain of financial security (β = 0.78, 95% CI 0.74, 0.82), and were lowest in the domain of emotional health (β = 0.51, 95% CI 0.44, 0.57). Each of the domains (except for character strengths as measured) was also positively associated with at least one other domain at the subsequent wave. For instance, those who reported greater emotional health also reported a higher level of physical health (β = 0.08, 95% CI 0.02, 0.14) and social connectedness (β = 0.07, 95% CI 0.01, 0.14) subsequently, after controlling for physical health and social connectedness at baseline (Table 2, Supplementary Figure S3). Likewise, there was evidence suggesting that physical health predicted subsequent emotional health, purpose and character strengths; meaning and purpose predicted subsequent emotional health, character strengths and social connectedness; social connectedness predicted subsequent emotional health, purpose and financial security; and financial security predicted subsequent emotional health, physical health, and social connectedness (Table 2, Supplementary Figures S4, S5, S6, S7 and S8).

Associations between other covariates and composite flourishing are also reported in the online supplement (Supplementary Table S6). When all covariates were simultaneously included in the models, not being a registered voter (vs. voted in the last presidential election) was the strongest predictor of lower levels of subsequent composite flourishing. When domains of flourishing were examined separately, not being a registered voter also predicted lower levels of subsequent emotional health and physical health. In addition, older age and volunteering were each associated with greater emotional health, and the number of physician-diagnosed physical health problems was inversely associated with subsequent physical health. Female gender and volunteering both predicted greater social connectedness; spiritual practices predicted greater character strengths; and spiritual practice and volunteering both predicted greater subsequent meaning and purpose. Black race, number of children, and the non-exempt work type were each inversely related to subsequent financial security, whereas homeownership was associated with greater subsequent financial security.

### Individual item indicators under each domain and subsequent flourishing

When individual items under the domain of emotional health were examined one at a time, multiple items were positively associated with composite flourishing subsequently (Table 3). When the items were examined simultaneously, only the two items assessing optimism (“I expect more good things in my life than bad”, β = 0.05, 95% CI 0.00, 0.10) and emotional regulation (“In stressful situations, I manage my emotions so that I am still in control of myself”, β = 0.06, 95% CI 0.01, 0.11) remained associated with composite flourishing. When the alternative composite flourishing score was considered as the dependent variable, none of these associations remained *p* < 0.05.

**Table 3:** Longitudinal associations between individual survey items of the flourishing index and subsequent flourishing (N = 1209^δ^).

Individual survey items of the flourishing index in 2018	Flourishing in 2019			
	Composite flourishing^±^	Composite flourishing (other domains only)^§^	Composite flourishing, with simultaneous control^σ^	Composite flourishing (other domains only), with simultaneous control^α^
**Emotional health domain**				
How satisfied are you with life as a whole these days?	0.04 (− 0.01, 0.09)	0.01 (− 0.04, 0.06)	0.01 (− 0.04, 0.06)	0.00 (− 0.05, 0.06)
How happy have you felt during the last 7 days?	0.03 (− 0.02, 0.08)	0.00 (− 0.05, 0.05)	− 0.02 (− 0.07, 0.04)	− 0.02 (− 0.07, 0.04)
I expect more good things in my life than bad	0.07 (0.02, 0.12)**	0.03 (− 0.01, 0.08)	0.05 (0.00, 0.10)*	0.03 (− 0.02, 0.08)
How would you rate your mental health?	0.09 (0.04, 0.14)***	0.03 (− 0.02, 0.08)	0.04 (− 0.02, 0.11)	0.02 (− 0.04, 0.08)
Are you depressed? (reversed)	0.07 (0.02, 0.11)**	0.01 (− 0.03, 0.05)	0.03 (− 0.02, 0.08)	0.00 (− 0.05, 0.05)
Do you have anxiety that keeps you from doing the things in life that you need to do? (reversed)	0.04 (0.01, 0.08)*	0.01 (− 0.03, 0.05)	0.01 (− 0.03, 0.05)	0.00 (− 0.04, 0.04)
In stressful situations, I manage my emotions so that I am still in control of myself	0.08 (0.04, 0.13)***	0.04 (0.00, 0.09)	0.06 (0.01, 0.11)**	0.04 (− 0.01, 0.08)
**Physical health domain**				
How would you rate your physical health?	0.06 (0.01, 0.10)*	− 0.02 (− 0.06, 0.03)	− 0.02 (− 0.07, 0.03)	− 0.05 (− 0.10, 0.00)
I have no major illnesses or injuries	0.11 (0.08, 0.15)***	0.04 (0.00, 0.08)	0.03 (− 0.02, 0.08)	0.02 (− 0.03, 0.07)
I do not routinely get sick	0.11 (0.07, 0.14)***	0.03 (− 0.01, 0.07)	0.03 (− 0.01, 0.08)	0.00 (− 0.05, 0.05)
My health does not prevent me from doing what I would like	0.12 (0.08, 0.16)***	0.03 (− 0.01, 0.07)	0.02 (− 0.04, 0.08)	0.01 (− 0.05, 0.06)
My pain makes it hard for me to do my usual activities. (reversed)	0.08 (0.04, 0.12)***	0.01 (− 0.03, 0.05)	0.02 (− 0.02, 0.07)	− 0.01 (− 0.06, 0.04)
Based on my past health, I expect to be healthy long into the future	0.16 (0.12, 0.20)***	0.06 (0.02, 0.10)**	0.09 (0.04, 0.15)**	0.06 (0.00, 0.12)*
I regularly do things to maintain and improve my health, in diet, exercise, and healthcare	0.08 (0.04, 0.13)***	0.02 (− 0.02, 0.06)	0.03 (− 0.01, 0.08)	0.01 (− 0.04, 0.06)
**Meaning and purpose domain**				
I have values and beliefs that help me understand who I am	0.06 (0.01, 0.11)*	0.01 (− 0.04, 0.06)	− 0.04 (− 0.09, 0.02)	− 0.05 (− 0.11, 0.00)
I know what gives meaning to my life	0.11 (0.06, 0.15)***	0.05 (0.00, 0.09)	0.05 (− 0.01, 0.11)	0.02 (− 0.04, 0.08)
My life has a clear sense of purpose	0.16 (0.11, 0.21)***	0.09 (0.04, 0.14)***	0.07 (− 0.03, 0.16)	0.06 (− 0.03, 0.15)
I understand my purpose in life	0.17 (0.12, 0.22)***	0.09 (0.05, 0.14)***	0.10 (0.02, 0.19)*	0.07 (− 0.02, 0.16)
To what extent do you feel the things you do in your life are worthwhile?	0.09 (0.04, 0.14)***	0.05 (0.00, 0.10)*	0.04 (− 0.01, 0.09)	0.03 (− 0.02, 0.08)
I am pursuing what is most important to me in my life	0.06 (0.01, 0.10)*	0.01 (− 0.04, 0.05)	− 0.03 (− 0.08, 0.03)	− 0.05 (− 0.10, 0.01)
**Character strengths domain**				
I always act to promote good in all circumstances, even in difficult and challenging situations	0.08 (0.03, 0.12)***	0.03 (− 0.02, 0.07)	0.08 (0.03, 0.14)**	0.07 (0.02, 0.12)**
I always know the right thing to do	0.00 (− 0.04, 0.04)	− **0.04 (**− 0.08, 0.00)*	− 0.03 (− 0.07, 0.02)	− **0.04 (**− 0.09, 0.00)*
I always treat everyone with kindness, fairness and respect	0.03 (− 0.01, 0.07)	− 0.02 (− 0.06, 0.02)	0.00 (− 0.05, 0.04)	− 0.03 (− 0.08, 0.01)
I am always able to give up some happiness now for greater happiness later	0.03 (− 0.01, 0.07)	− 0.01 (− 0.05, 0.02)	0.01 (− 0.03, 0.06)	0.00 (− 0.05, 0.05)
I am willing to face difficulties in order to do what is right	0.02 (− 0.02, 0.06)	− 0.02 (− 0.06, 0.02)	− 0.01 (− 0.06, 0.04)	− 0.02 (− 0.07, 0.03)
I give up personal pleasures whenever it is possible to do some good instead	0.02 (− 0.02, 0.06)	− 0.02 (− 0.06, 0.02)	0.00 (− 0.05, 0.05)	− 0.01 (− 0.06, 0.04)
I get to use my strengths to help others	0.01 (− 0.03, 0.05)	− 0.02 (− 0.06, 0.02)	0.00 (− 0.05, 0.04)	− 0.01 (− 0.06, 0.03)
**Social connectedness domain**				
My relationships are as satisfying as I would want them to be	0.11 (0.06, 0.16)***	0.05 (0.00, 0.09)*	0.04 (− 0.02, 0.10)	0.03 (− 0.03, 0.09)
There are people who really understand me	0.11 (0.06, 0.15)***	0.05 (0.01, 0.09)*	0.04 (− 0.01, 0.10)	0.03 (− 0.02, 0.08)
How often do you feel lonely? (reversed)	0.06 (0.01, 0.10)*	0.02 (− 0.03, 0.06)	0.02 (− 0.03, 0.07)	0.00 (− 0.04, 0.05)
I am content with my friendships and relationships	0.12 (0.07, 0.16)***	0.04 (0.00, 0.09)	0.02 (− 0.05, 0.09)	− 0.01 (− 0.08, 0.06)
I have enough people I feel comfortable asking for help at any time	0.11 (0.07, 0.16)***	0.05 (0.01, 0.09)*	0.06 (0.01, 0.11)*	0.04 (− 0.02, 0.09)
I feel connected to the broader community around me	0.08 (0.03, 0.12)**	0.02 (− 0.03, 0.06)	0.00 (− 0.07, 0.06)	− 0.02 (− 0.08, 0.04)
People in my broader community trust and respect one another	0.07 (0.02, 0.11)**	0.02 (− 0.02, 0.06)	0.03 (− 0.03, 0.08)	0.01 (− 0.05, 0.07)
**Financial security domain**				
I am able to meet my normal monthly living expenses without any difficulty	0.25 (0.20, 0.29)***	0.07 (0.02, 0.11)**	0.09 (0.03, 0.15)**	0.04 (− 0.02, 0.11)
How often do you worry about food, housing, or health expenses? (reversed)	0.20 (0.16, 0.24)***	0.05 (0.00, 0.09)*	0.04 (− 0.01, 0.09)	0.00 (− 0.06, 0.06)
I have sufficient savings that I could cover six months of expenses	0.26 (0.22, 0.30)***	0.06 (0.02, 0.10)**	0.11 (0.05, 0.18)**	0.03 (− 0.04, 0.10)
My financial circumstances give me freedom to pursue my goals	0.24 (0.20, 0.29)***	0.05 (0.00, 0.09)*	− 0.01 (− 0.08, 0.07)	− 0.03 (− 0.11, 0.04)
Given my age, I have done adequate financial planning for the future	0.24 (0.20, 0.28)***	0.06 (0.01, 0.11)**	0.08 (0.02, 0.14)*	0.03 (− 0.03, 0.10)
The amount of debt I have often overwhelms me	0.21 (0.17, 0.26)***	0.05 (0.01, 0.10)*	0.06 (0.01, 0.11)*	0.02 (− 0.04, 0.08)

A set of separate linear regression models were used to regress the outcome variables at follow-up (one at a time) on individual items under each flourishing domain at baseline (these individual items were first examined separately one at a time, and then were included simultaneously in the models), adjusting for the other 5 domain-specific scores that were not used as the independent variable. All models adjusted for gender, age, race, marital status, voting, education, number of children, care for older persons, home ownership, religious service attendance, spirituality, community participation, volunteering, work hours, salary bands, work type and the number of physician-diagnosed physical health problems. The domain of physical health in the flourishing index assessed self-rated health, whereas the covariate of physician-diagnosed illness was an objective indicator of health. Likewise, the domain of social connectedness in the flourishing index assessed perceived social relationships, whereas the covariates of social participation measured objective features of the social networks.

Bolded values indicate: *p < .05 before Bonferroni correction, **p < *.*01 before Bonferroni correction, ***p < *.*05 after Bonferroni correction (the *p* value cutoff for Bonferroni correction is p = 0.05/92 tests = 0.0005).

^δ^Participants with missing data on the dependent variables were excluded from the analyses. Multiple imputation was performed to impute missing data on the independent variables and covariates.

^±^The models examined the associations between individual items of the flourishing index in 2018 (*examined one at a time*) and a composite flourishing score in 2019 that *includes all flourishing domains*.

^§^The models examined the associations between individual items of the flourishing index in 2018 (*examined one at a time*) and an alternative composite flourishing score in 2019 that *excludes the particular domain under which the items were used as the independent variables.*

^σ^The models examined the associations between individual items of the flourishing index in 2018 (items under each domain were simultaneously included in the models) and a composite flourishing score in 2019 that *includes all flourishing domains*.

^α^The models examined the associations between individual items of the flourishing index in 2018 (items under each domain were simultaneously included in the models) and an alternative composite flourishing score in 2019 that *excludes the particular domain under which the items were used as the independent variables.*

All items under the domain of physical health, when examined separately, were positively associated with subsequent composite flourishing. However, the only association that remained robust across other analyses was with the item: “Based on my past health, I expect to be healthy long into the future” (e.g., β = 0.06, 95% CI 0.00, 0.12, in the analysis that examined all items simultaneously and used the alternative flourishing score) (Table 3).

When examined separately, all items under the domain of meaning and purpose were positively associated with subsequent composite flourishing. However, only one item (“I understand my purpose in life”) remained associated when the items were examined simultaneously (β = 0.10, 95% CI 0.02, 0.19). Likewise, multiple items were associated with the alternative composite flourishing score when examined separately, but none of the associations held when the items were examined simultaneously (Table 3).

Under the domain of character strengths, the item “I always act to promote good in all circumstances, even in difficult and challenging situations” was positively related to subsequent composite flourishing (e.g., β = 0.07, 95% CI 0.02, 0.12, with the alternative flourishing score as the dependent variable, when all items were controlled simultaneously). Interestingly, this was also one of the two items, out of all 40 items, that had the most robust associations with composite flourishing when simultaneous control was made for other indicators. In the individual indicator analyses, another character item “I always know the right thing to do” was inversely associated with subsequent flourishing when the alternative flourishing score was considered (e.g., β = − 0.04, 95% CI − 0.09, 0.00, with all items controlled simultaneously) (Table 3).

Multiple items under the domain of social connectedness, when examined separately, were positively associated with subsequent composite flourishing. However, the only association that remained robust when the items were examined simultaneously was with: “I have enough people I feel comfortable asking for help at any time” (β = 0.06, 95% CI 0.01, 0.11, in relation to the alternative composite flourishing score) (Table 3).

Multiple items under the financial security domain were positively associated with subsequent composite flourishing. The item that showed the most robust association was “I have sufficient savings that I could cover six months of expenses” (e.g., β = 0.11, 95% CI 0.05, 0.18, when the items were examined simultaneously). All items were associated with the alternative composite flourishing score when examined separately, whereas none of these associations remained when the items were examined simultaneously (Table 3).

Results of the secondary analyses on domain-specific scores and subsequent well-being on individual item indicators of flourishing are shown in Supplementary Tables S7 and S8. The associations between individual survey items and subsequent domain-specific well-being scores are provided in Supplementary Tables S9A, S9B, S10A, S10B, S11A, S11B, S12A, S12B, S13A, S13B, S14A, and S14B.

## Discussion

This study provided evidence on the longitudinal associations between domains of flourishing based on the broad theoretical framework of human flourishing as proposed by VanderWeele^1^. The results suggested that all domains were each independently associated with greater composite flourishing and with greater well-being in at least one domain other than itself subsequently, although with the weakest evidence for character strengths. The effect sizes are generally modest across domains over the one-year follow-up, but even small effect sizes could have significant implications at the population level, especially if the effects accumulate over time^55^.

Congruent with prior work, this study found bidirectional associations between emotional health and physical health, and between purpose and social connectedness. Specifically, a large body of prior work has suggested associations between indicators of psychological and mental well-being and subsequent physical health^56^. For instance, a few recent review studies^16,57,58^ indicated that multiple facets of psychological well-being (e.g., optimism, emotional vitality, life satisfaction) were associated with greater cardiovascular health subsequently, possibly through shaping neurobiological processes, health behaviors, or other psychosocial resources that in turn promote health or buffer stress^16^. On the other hand, cardiovascular health or related health behaviors (e.g., smoking) may also influence subsequent psychological well-being^15,59^, possibly through imposing physical limitations that influence individuals’ opportunities for engaging in activities (e.g., socializing) that confer positive feelings or satisfaction^60^. Likewise, prior studies have also suggested that purpose in life and social connectedness may shape and strengthen each other in a positive cycle^61^. For instance, multiple facets of social connectedness such as frequent contacts with friends and having a sense of belonging to a larger community have been linked to a greater sense of meaning and purpose^61,62^. It was hypothesized that having close social relationships may intrinsically motivate helping others, which contributes to a sense of being useful and respected^63^. Conversely, individuals who reported greater meaning in life were more likely to consider having close social relationships as important life goals^64^, and were more likely to develop stable social relationships^61^.

In comparison, this study found weaker associations of purpose and social connectedness in relation to physical health, as compared to some prior work. Specifically, somewhat contrary to the growing evidence suggesting that greater purpose in life was associated with better physical health^30,31^ and healthier behaviors^65^, this study did not find strong evidence for such associations. However, prior work used objective measures of health (e.g., mortality) and had longer follow-up periods^22^. Such contrary findings might also be attributed to differences in the measurement of purpose – the scale used to assess purpose in this study comprised a mixture of items assessing both meaning and purpose, whereas most prior work measured purpose more specifically^66^. Interestingly, consistent with some prior findings^67^, when individual items under the domain of meaning and purpose were examined separately in this study, the items assessing purpose were more strongly associated with physical health than those measuring meaning. Likewise, prior studies suggested associations between multiple indicators of social integration and better subsequent physical health^68,69^, whereas this study did not find such associations. However, it is also possible, both for purpose and with social connectedness, that the effects of these on physical health may need a longer time to accumulate and thus were not captured by our short-term follow-up of only one year. Moreover, the scale for assessing social connectedness in this study focused on assessing relationship satisfaction and relationship quality, whereas prior studies often measured frequency of contacts with social ties^70^. In the individual indicator analyses the most robustly associated social item was “I have enough people I feel comfortable asking for help at any time”.

In philosophical traditions, virtue and character strengths are considered an essential constituent of a flourishing life^12,13^. Some prior empirical studies on specific traits of character strengths suggested associations between happiness-related strengths (e.g., hope, love, gratitude) and greater subsequent psychological well-being^33,71,72^. In comparison, this study assessed character strengths with more generic measures of global character strengths. While this study found little evidence that character strengths, as assessed by the present items, were related with subsequent well-being in other domains of flourishing, several things should be held in mind. First, it may be more difficult to self-report on character strengths than on other domains of flourishing due to social desirability or a lack of self-awareness; alternatively, it may also be the case that the these newly proposed items are not the best item formulations for the purposes of self-assessment^73^. This may have been the case for example with the item “I always know the right thing to do,” which had negative associations with subsequent flourishing. It is possible that for some individuals “always knowing the right thing to do” may indicate an overestimation of one’s own abilities and a lack of motivation for learning or self-reflection, which may impede one’s subsequent flourishing. Second, it is possible that the present analyses over-control for prior effects of character on well-being by simultaneously controlling for other aspects of well-being at baseline and also controlling for variables that are associated with character—and indeed have been used in some research as markers for character and virtue—such as volunteering, care for older persons, or spirituality^74^. Third, while the domain as a whole was not strongly predictive, one of the items, “I always act to promote good in all circumstances, even in difficult and challenging situations” was strongly predictive of subsequent composite flourishing. This indicator of character has likewise been strongly predictive of well-being outcomes in other studies^75,76^. In this study, that indicator was one of the only 2 indicators (out of 40) that was predictive of composite flourishing in other domains when controlling for other indicators (final column in Table 3). Finally, it is possible that exercising character strengths may not be strongly associated with one’s own health and well-being, but may benefit other people’s well-being and help promote community flourishing. Nevertheless, the weak association with other subsequent domains of flourishing for all but one of the character indicators should give some hesitation about using these items in future studies, at least until more is understood about self-assessment when evaluating character strengths.

This last point leads to other interesting considerations with regard to the item-by-item analyses in Table 3. While the items were developed to assess domains of flourishing, the analyses presented in Table 3 may be informative with regard to what items one might want to assess if survey space constraints are more limited. The associations reflect the predictive strength of each individual indicator on subsequent well-being. Several observations in this regard are quite striking. With regard to emotional health, once simultaneous control is made for all emotional indicators, optimism (“I expect more good things in my life than bad”) and emotion regulation (“In stressful situations, I manage my emotions so that I am still in control of myself”) are most predictive of subsequent well-being. The life satisfaction indicator (“How satisfied are you with life as a whole these days?”), which is one of the most commonly employed in the well-being literature, is not particularly predictive in this study. This does not mean that it is necessarily a bad item; viewed as an outcome, it may be considered, by many, to be very important; but it is not especially predictive of leading to higher well-being in the future in this sample. With respect to physical health, the responses to the item “Based on my past health, I expect to be healthy long into the future” were most predictive, though this may reflect both physical health and optimism. In any case, this item was notably more predictive than the standard self-rated health question. In the meaning and purpose domain, the responses to the items that made direct reference to “purpose” (“My life has a clear sense of purpose” and “I understand my purpose in life”) were most predictive, and more so than those oriented more towards meaning. As noted above, the only strongly predictive indicator in the character domain was “I always act to promote good in all circumstances, even in difficult and challenging situations,” and this was one of the most robust among the whole set of 40 items. In the social connectedness domain, one indicator related to social support, “I have enough people I feel comfortable asking for help at any time”, was most predictive. In the financial security domain, several indicators were similarly predictive: “I am able to meet my normal monthly living expenses without any difficulty”, “I have sufficient savings that I could cover six months of expenses”, “My financial circumstances give me freedom to pursue my goals”, and “Given my age, I have done adequate financial planning for the future”.

For shorter well-being assessments, these results may provide some guidance as to what items to use. A number of these are included in the shorter 12-item flourishing index^1^, though this was formed prior to the present analyses. However, as noted above, the predictive ability of these indicators with respect to increases in future well-being is not the only relevant criterion in determining what to include in a well-being index; many—indeed most—of the single-item indicators of well-being here are not of value simply because of contributing to future well-being but because they are sought in and of themselves.

This study has certain limitations. First, participants of this study were self-selected working employees and the retention rate at the subsequent wave was not ideal. Results of this study, therefore, may not be generalizable to other populations. Second, the participants were predominantly female (the gender ratio in this sample was in fact roughly representative of the whole employee population in this organization which was large majority female), and the results may primarily reflect the associations in women. It would be helpful to examine potential gender differences in samples with more equal representation of gender in future studies. Next, the follow-up period in this study was relatively short, which might not be able to capture longer-term dynamics between some domains of flourishing. Participants in this sample were mostly in their early to middle-adulthood and were relatively healthy, and follow-up was only for 1 year. The influences of some domains such as purpose or character strengths on physical health may thus take a longer time to accumulate and unfold. These limitations were, however, balanced by important strengths of this study including longitudinal data, rich covariate control, and a 40-item well-being assessment.

There has been growing interest worldwide in measuring flourishing as an additional approach for monitoring and promoting holistic well-being^2,8^. Further understandings of the constituents of a flourishing life and their longitudinal interrelationships would help inform social programs and policy initiatives to promoting population health and well-being in its fullest sense.

## Supplementary Information

Supplementary Information 1.

Supplementary Information 2.
