# Supplementary material for: Longitudinal associations between domains of flourishing
Source: Sci Rep. 2022 Feb 17;12:2740. doi: 10.1038/s41598-022-06626-5 (PMC8854559; doi:10.1038/s41598-022-06626-5)
Supplement: Supplementary file 1 — Supplementary Information 1. [file 41598_2022_6626_MOESM1_ESM.docx]

**ONLINE SUPPLEMENTARY MATERIALS**

This online supplement provides additional information for the study “Longitudinal associations between domains of flourishing”.

| **Supplementary Table S1. Baseline characteristics of participants who remained in the cohort and participants who dropped out at follow-up (N=2,364).** | | | | |
| --- | --- | --- | --- | --- |
| **Participant characteristics** | **Participants who stayed in the cohort (n=1,411)** | **Participants who dropped out at follow-up (n=953)** | **Cramer’s V** | ***P*-value** |
| Age categories, % |  |  | 0.082 | .001 |
| 30 years or below | 11.27 | 16.68 |  |  |
| 31-40 years | 28.49 | 29.07 |  |  |
| 41-50 years | 29.91 | 27.07 |  |  |
| 50+ years | 30.33 | 27.18 |  |  |
| Gender, % |  |  | 0.060 | .004 |
| Female | 84.27 | 79.64 |  |  |
| Male | 15.73 | 20.36 |  |  |
| Race/Ethnicity, % |  |  | 0.079 | <.001 |
| Non-Hispanic White | 75.05 | 67.89 |  |  |
| Black/African American | 11.48 | 15.01 |  |  |
| Others | 13.47 | 17.10 |  |  |
| Marital status, % |  |  | 0.084 | .005 |
| Single | 15.95 | 18.37 |  |  |
| Married | 61.85 | 54.03 |  |  |
| Divorced | 11.06 | 13.64 |  |  |
| Widowed | 1.44 | 1.18 |  |  |
| Separated | 1.29 | 2.15 |  |  |
| Non-married partner | 8.41 | 10.63 |  |  |
| Educational attainment, % |  |  | 0.022 | .89 |
| High school diploma or equivalent | 7.52 | 6.86 |  |  |
| Some college but not degree | 22.55 | 23.26 |  |  |
| Associate degree | 13.53 | 13.18 |  |  |
| Bachelor degree | 35.79 | 37.19 |  |  |
| Graduate degree | 20.62 | 19.51 |  |  |
| House ownership, % | 72.88 | 67.35 | 0.060 | .004 |
| Work hours (hours/day), % |  |  | 0.013 | 1.00 |
| <8 | 1.14 | 1.07 |  |  |
| 8 | 49.50 | 49.63 |  |  |
| 9-10 | 36.70 | 36.04 |  |  |
| 11-12 | 6.72 | 6.84 |  |  |
| 13-14 | 0.57 | 0.53 |  |  |
| >14 | 5.36 | 5.88 |  |  |
| Number of children under the age of 18 years |  |  | 0.022 | .94 |
| 0 | 52.19 | 53.70 |  |  |
| 1 | 20.36 | 20.36 |  |  |
| 2 | 18.28 | 16.61 |  |  |
| 3 | 6.67 | 6.86 |  |  |
| 4 | 1.86 | 1.93 |  |  |
| 5+ | 0.65 | 0.54 |  |  |
| Taking care of older persons, % | 20.84 | 17.76 | 0.038 | .08 |
| Frequency of religious service attendance, % |  |  | 0.040 | .45 |
| Daily or more than once a week | 5.87 | 4.28 |  |  |
| Once a week | 14.39 | 14.13 |  |  |
| 1-3 times a month | 11.74 | 12.10 |  |  |
| Once every few months or once a year | 39.58 | 38.97 |  |  |
| Never | 28.42 | 30.51 |  |  |
| Frequency of spiritual practice, % |  |  | 0.040 | .61 |
| Daily | 27.73 | 29.05 |  |  |
| Not daily but more than once a week | 24.80 | 22.51 |  |  |
| Once a week | 8.36 | 7.93 |  |  |
| 1-3 times a month | 12.51 | 13.61 |  |  |
| Once every few months or once a year | 18.23 | 17.26 |  |  |
| Never | 8.36 | 9.65 |  |  |
| Frequency of participation in community groups, % |  |  | 0.047 | .31 |
| Daily or more than once a week | 1.02 | 1.96 |  |  |
| Once a week | 9.14 | 8.54 |  |  |
| 1-3 times a month | 17.02 | 18.57 |  |  |
| Once every few months or once a year | 37.59 | 35.41 |  |  |
| Never | 35.22 | 35.52 |  |  |
| Frequency of volunteering, % |  |  | 0.032 | .67 |
| Daily or more than once a week | 4.73 | 3.85 |  |  |
| Once a week | 4.37 | 3.53 |  |  |
| 1-3 times a month | 13.19 | 13.60 |  |  |
| Once every few months or once a year | 50.18 | 50.21 |  |  |
| Never | 27.53 | 28.80 |  |  |
| Voted in the last presidential election, % |  |  | 0.043 | .23 |
| Yes | 82.69 | 79.72 |  |  |
| No | 13.88 | 16.20 |  |  |
| Not a registered voter | 3.43 | 4.08 |  |  |

Note: Chi-square tests were used to compare the groups in this table.

Data on salary bands was available only for those who participated in both waves of data collection in this study. Therefore, the variable salary bands were not available in this table due to lack of data for the dropouts.

| **Supplementary Table S2. The 40-item flourishing index** | |
| --- | --- |
| **Individual Survey Items** | **Response Options (All on a 0-10 Scale)** |
| ***Emotional health*** |  |
| How satisfied are you with life as a whole these days? | 0 = Not Satisfied at All, 10 = Completely Satisfied |
| How happy have you felt during the last 7 days? | 0 = Extremely Unhappy, 10 = Extremely Happy |
| I expect more good things in my life than bad. | 0=Strongly Disagree, 10=Strongly Agree |
| How would you rate your mental health? | 0 = Poor, 10 = Excellent |
| Are you depressed? (reversed) | 0=Not at all Depressed, 10=Very Depressed |
| Do you have anxiety that keeps you from doing the things in life that you need to do? (reversed) | 0=Never, 10=Always |
| In stressful situations, I manage my emotions so that I am still in control of myself. | 0=Strongly Disagree, 10=Strongly Agree |
|  |  |
| ***Physical Health*** |  |
| How would you rate your physical health? | 0 = Poor, 10 = Excellent |
| I have no major illnesses or injuries. | 0=Strongly Disagree, 10=Strongly Agree |
| I do not routinely get sick. | 0=Strongly Disagree, 10=Strongly Agree |
| My health does not prevent me from doing what I would like. | 0=Strongly Disagree, 10=Strongly Agree |
| My pain makes it hard for me to do my usual activities. (reversed) | 0=Strongly Disagree, 10=Strongly Agree |
| Based on my past health, I expect to be healthy long into the future. | 0=Strongly Disagree, 10=Strongly Agree |
| I regularly do things to maintain and improve my health, in diet, exercise, and healthcare. | 0=Strongly Disagree, 10=Strongly Agree |
|  |  |
| ***Meaning and purpose*** |  |
| I have values and beliefs that help me understand who I am. | 0=Strongly Disagree, 10=Strongly Agree |
| I know what gives meaning to my life. | 0=Strongly Disagree, 10=Strongly Agree |
| My life has a clear sense of purpose. | 0=Strongly Disagree, 10=Strongly Agree |
| I understand my purpose in life. | 0=Strongly Disagree, 10=Strongly Agree |
| To what extent do you feel the things you do in your life are worthwhile? | 0 = Not at All Worthwhile, 10 = Completely Worthwhile |
| I am pursuing what is most important to me in my life. | 0=Strongly Disagree, 10=Strongly Agree |
|  |  |
| ***Character Strengths*** |  |
| I always act to promote good in all circumstances, even in difficult and challenging situations. | 0=Not True of Me; 10=Completely True of Me |
| I always know the right thing to do. | 0=Not True of Me; 10=Completely True of Me |
| I always treat everyone with kindness, fairness and respect. | 0=Not True of Me; 10=Completely True of Me |
| I am always able to give up some happiness now for greater happiness later. | 0=Not True of Me; 10=Completely True of Me |
| I am willing to face difficulties in order to do what is right. | 0=Not True of Me; 10=Completely True of Me |
| I give up personal pleasures whenever it is possible to do some good instead. | 0=Not True of Me; 10=Completely True of Me |
| I get to use my strengths to help others. | 0=Not True of Me; 10=Completely True of Me |
|  |  |
| ***Social Connectedness*** |  |
| My relationships are as satisfying as I would want them to be. | 0=Strongly Disagree, 10=Strongly Agree |
| There are people who really understand me. | 0=Never, 10=Often |
| How often do you feel lonely? (reversed) | 0=Never,10=Almost Always |
| I am content with my friendships and relationships. | 0=Strongly Disagree, 10=Strongly Agree |
| I have enough people I feel comfortable asking for help at any time. | 0=Strongly Disagree, 10=Strongly Agree |
| I feel connected to the broader community around me. | 0=Strongly Disagree, 10=Strongly Agree |
| People in my broader community trust and respect one another. | 0=Strongly Disagree, 10=Strongly Agree |
|  |  |
| ***Financial Security*** |  |
| I am able to meet my normal monthly living expenses without any difficulty. | 0=Completely Disagree, 10=Completely Agree |
| How often do you worry about food, housing, or health expenses? (reversed) | 0=Do Not Ever Worry, 10=Worry All of the Time |
| I have sufficient savings that I could cover six months of expenses. | 0=Strongly Disagree, 10=Strongly Agree |
| My financial circumstances give me freedom to pursue my goals. | 0=Strongly Disagree, 10=Strongly Agree |
| Given my age, I have done adequate financial planning for the future. | 0=Strongly Disagree, 10=Strongly Agree |
| The amount of debt I have often overwhelms me. (reversed) | 0=Strongly Disagree, 10=Strongly Agree |

| **Supplementary Table S3. Cronbach’s alpha of the scales measuring domains of flourishing at both waves (N=1,209).** | | |
| --- | --- | --- |
| **Flourishing domains** | **Cronbach’s alpha** | |
|  | Baseline (2018) | Follow-up (2019) |
| Emotional health | 0.88 | 0.90 |
| Physical health | 0.87 | 0.90 |
| Meaning and Purpose | 0.93 | 0.95 |
| Character strengths | 0.86 | 0.90 |
| Social connectedness | 0.90 | 0.92 |
| Financial security | 0.92 | 0.93 |

| **Supplementary Table S4. Descriptive statistics on the distribution of flourishing scores at study baseline and at follow-up (N=1,209)** | | |
| --- | --- | --- |
| **Flourishing scores, mean (SD)** | **At baseline** | **At follow-up** |
| Composite Flourishing Score | 7.48 (1.29) | 7.70 (1.34) |
| Emotional health | 7.56 (1.56) | 7.65 (1.55) |
| Physical health | 7.82 (1.66) | 8.07 (1.67) |
| Meaning and purpose | 7.98 (1.53) | 8.12 (1.55) |
| Character strengths | 7.92 (1.17) | 8.19 (1.16) |
| Social connectedness | 7.36 (1.75) | 7.59 (1.72) |
| Financial security | 6.31 (2.64) | 6.56 (2.67) |

Notes: The composite flourishing score and all domain-specific scores were all measured on a 0-10 scale.

**Supplementary Figure S1. Longitudinal associations between domains of flourishing and subsequent composite flourishing.**

**Supplementary Figure S2. Longitudinal associations between domains of flourishing and subsequent composite flourishing (alternative score+).**

**Supplementary Figure S3. Longitudinal associations between domains of flourishing and subsequent emotional health.**

**Supplementary Figure S4. Longitudinal associations between domains of flourishing and subsequent physical health.**

**Supplementary Figure S5. Longitudinal associations between domains of flourishing and subsequent meaning and purpose.**

**Supplementary Figure S6. Longitudinal associations between domains of flourishing and subsequent character strengths.**

**Supplementary Figure S7. Longitudinal associations between domains of flourishing and subsequent social connectedness.**

**Supplementary Figure S8. Longitudinal associations between domains of flourishing and subsequent financial security.**

| **Supplementary Table S5. Complete-case analyses on the longitudinal associations between domains of flourishing (N=1,014).** | | | | | | | | | |
| --- | --- | --- | --- | --- | --- | --- | --- | --- | --- |
| **2019 Flourishing** | | | | | | | | | |
| **2018 Flourishing domains** | Emotional  health | Physical health | Meaning  and purpose | Character strengths | Social  connectedness | Financial security |  | Composite  flourishing | Composite flourishing (alternative)± |
| Emotional health | **0.52*****  **(0.45, 0.59)** | **0.08***  **(0.01, 0.14)** | 0.00  (-0.08, 0.07) | 0.01  (-0.07, 0.08) | 0.06  (-0.01, 0.13) | 0.00  (-0.06, 0.05) |  | **0.13*****  **(0.07, 0.19)** | 0.03  (-0.03, 0.09) |
| Physical health | 0.04  (-0.02, 0.09) | **0.67*****  **(0.61, 0.72)** | 0.05  (-0.01, 0.11) | **0.07***  **(0.01, 0.13)** | 0.01  (-0.04, 0.07) | -0.01  (-0.06, 0.03) |  | **0.17*****  **(0.12, 0.21)** | 0.03  (-0.02, 0.08) |
| Meaning and purpose | 0.06  (-0.02, 0.14) | 0.01  (-0.07, 0.08) | **0.59*****  **(0.51, 0.66)** | **0.12****  **(0.04, 0.20)** | **0.11****  **(0.03, 0.19)** | 0.01  (-0.05, 0.07) |  | **0.17*****  **(0.10, 0.24)** | **0.07***  **(0.00, 0.13)** |
| Character strengths | 0.00  (-0.06, 0.06) | -0.03  (-0.09, 0.02) | -0.01  (-0.07, 0.06) | **0.54*****  **(0.48, 0.60)** | -0.03  (-0.09, 0.03) | -0.02  (-0.07, 0.03) |  | **0.06***  **(0.01, 0.11)** | -0.02  (-0.07, 0.03) |
| Social connectedness | **0.08***  **(0.01, 0.15)** | 0.02  (-0.04, 0.09) | **0.09***  **(0.02, 0.16)** | -0.01  (-0.08, 0.06) | **0.57*****  **(0.50, 0.63)** | **0.05***  **(0.00, 0.11)** |  | **0.17*****  **(0.11, 0.23)** | **0.06***  **(0.00, 0.12)** |
| Financial security | **0.10*****  **(0.04, 0.15)** | **0.08****  **(0.03, 0.14)** | **0.07***  **(0.01, 0.12)** | **0.07***  **(0.01, 0.13)** | **0.07****  **(0.02, 0.13)** | **0.78*****  **(0.74, 0.83)** |  | **0.34*****  **(0.29, 0.38)** | **0.09*****  **(0.04, 0.15)** |
| Note: All models adjusted for gender, age, race, marital status, voting, education, number of children, care for older persons, home ownership, religious service attendance, spirituality, community participation, volunteering, work hours, salary bands, work type, number of physician-diagnosed physical health problems, and all 2018 flourishing domains simultaneously. The domain of physical health in the flourishing index assessed self-rated health, whereas the covariate of physician-diagnosed illness was an objective indicator of health. Likewise, the domain of social connectedness in the flourishing index assessed perceived social relationships, whereas the covariates of social participation measured objective features of social networks.  ± An alternative composite flourishing score excluding the specific domain taken as the independent variable in each model.  **p*<.05 before Bonferroni correction, ***p<.*01 before Bonferroni correction, ****p<.*05 after Bonferroni correction (the p value cutoff for Bonferroni correction is p=0.05/48 tests=0.001). | | | | | | | | | |

| **Supplementary Table S6. Associations between all covariates and subsequent composite flourishing (N=1,209^⸹^).** | | | |
| --- | --- | --- | --- |
| **Covariates** | **ß (standardized)+** | **95% Confidence interval** | ***P*-value** |
| Emotional health | 0.13 | 0.07, 0.19 | <.0001 |
| Physical health | 0.17 | 0.13, 0.22 | <.0001 |
| Social connectedness | 0.17 | 0.12, 0.22 | <.0001 |
| Character strengths | 0.05 | 0.00, 0.10 | 0.051 |
| Financial security | 0.32 | 0.28, 0.37 | <.0001 |
| Meaning and Purpose | 0.19 | 0.13, 0.25 | <.0001 |
| Race/ethnicity (Non-Hispanic White as ref) |  |  |  |
| Black/African-American | -0.06 | -0.17, 0.06 | 0.3637 |
| Other race/ethnicity | 0.07 | -0.04, 0.17 | 0.2283 |
| Age categories (<=30 years as ref) |  |  |  |
| 31-40 years | -0.08 | -0.21, 0.05 | 0.2369 |
| 41-50 years | -0.02 | -0.16, 0.11 | 0.7345 |
| 50+ years | 0.08 | -0.07, 0.23 | 0.2996 |
| Male (female as ref) | -0.03 | -0.13, 0.07 | 0.5607 |
| Marital status (single as ref) |  |  |  |
| Married | -0.01 | -0.12, 0.11 | 0.9213 |
| Divorced | -0.02 | -0.17, 0.13 | 0.7523 |
| Widowed | 0.06 | -0.24, 0.37 | 0.6834 |
| Separated | 0.08 | -0.25, 0.41 | 0.6412 |
| Non-married partner | -0.06 | -0.21, 0.09 | 0.4402 |
| Voting registration status (yes as ref) |  |  |  |
| No | -0.04 | -0.14, 0.06 | 0.4749 |
| Not a register voter | -0.23 | -0.42, -0.05 | 0.0145 |
| Educational attainment (high school diploma or equivalent as ref) |  |  |  |
| Some college but no degree | 0.05 | -0.09, 0.19 | 0.5013 |
| Associate degree | -0.03 | -0.19, 0.12 | 0.6663 |
| Bachelor degree | -0.07 | -0.22, 0.07 | 0.31 |
| Graduate school | -0.07 | -0.23, 0.08 | 0.3727 |
| Home ownership (no as ref) | 0.04 | -0.05, 0.14 | 0.3892 |
| Number of children (0 as ref) |  |  |  |
| 1 | -0.01 | -0.10, 0.09 | 0.8923 |
| 2 | -0.02 | -0.13, 0.09 | 0.7272 |
| 3 | -0.06 | -0.21, 0.09 | 0.4428 |
| 4 | -0.12 | -0.40, 0.16 | 0.3873 |
| 5+ | 0.06 | -0.34, 0.47 | 0.7655 |
| Take care of elders at home (no as ref) | 0.04 | -0.04, 0.12 | 0.2745 |
| Religious service attendance (never as ref) |  |  |  |
| Daily or more than once a week | 0.10 | -0.08, 0.27 | 0.2818 |
| Once a week | -0.09 | -0.21, 0.04 | 0.1759 |
| 1-3 times a month | 0.02 | -0.11, 0.15 | 0.7773 |
| Once every few months or once a year | -0.02 | -0.11, 0.08 | 0.7427 |
| Spirituality (never as ref) |  |  |  |
| Daily | 0.08 | -0.08, 0.23 | 0.3386 |
| Not daily but more than once a week | 0.11 | -0.04, 0.26 | 0.1474 |
| Once a week | 0.02 | -0.15, 0.20 | 0.7997 |
| 1-3 times a month | 0.11 | -0.04, 0.27 | 0.1591 |
| Once every few months or once a year | 0.07 | -0.08, 0.22 | 0.3745 |
| Community participation (never as ref) |  |  |  |
| Daily or more than once a week | 0.00 | -0.14, 0.14 | 0.9971 |
| Once a week | 0.07 | -0.07, 0.21 | 0.3305 |
| 1-3 times a month | 0.00 | -0.12, 0.12 | 0.9925 |
| Once every few months or once a year | 0.01 | -0.09, 0.10 | 0.9061 |
| Volunteering (never as ref) |  |  |  |
| Daily or more than once a week | 0.14 | -0.05, 0.32 | 0.1448 |
| Once a week | 0.16 | -0.02, 0.34 | 0.0819 |
| 1-3 times a month | 0.09 | -0.04, 0.21 | 0.1881 |
| Once every few months or once a year | 0.04 | -0.05, 0.13 | 0.3687 |
| Work hours (<8 hours as ref) |  |  |  |
| 8 hours | -0.01 | -0.32, 0.30 | 0.9652 |
| 9-10 hours | -0.01 | -0.33, 0.30 | 0.9404 |
| 11-12 hours | 0.08 | -0.26, 0.42 | 0.6496 |
| 13-14 hours | -0.09 | -0.65, 0.46 | 0.7348 |
| >14 hours | 0.01 | -0.33, 0.35 | 0.962 |
| Non-exempt work type (exempt as ref) | -0.09 | -0.19, 0.01 | 0.0855 |
| Salary bands | -0.01 | -0.06, 0.04 | 0.5929 |
| Number of physician-diagnosed health conditions | -0.02 | -0.06, 0.02 | 0.3783 |

^⸹^  Participants with missing data on the outcome (composite flourishing) were excluded from the analyses. Multiple imputation was performed to impute missing data on the independent variables and other covariates.

**+** The outcome (composite flourishing) and all continuous predictors were standardized at mean=0 and standard deviation=1.

| **Supplementary Table S7. The longitudinal associations between domains of flourishing and subsequent well-being on individual survey items of flourishing (N=1,209**^⸹^**).** | | | | | | |
| --- | --- | --- | --- | --- | --- | --- |
| **2018 Flourishing domains** | | | | | | |
| **2019 Flourishing survey items** | Emotional  health | Physical  health | Meaning and purpose | Character strengths | Social  connectedness | Financial  security |
| ***Emotional health*** |  |  |  |  |  |  |
| How satisfied are you with life as a whole these days? | **0.25*****  **(0.17, 0.32)** | **0.07***  **(0.01, 0.13)** | **0.17*****  **(0.09, 0.25)** | -0.04  (-0.10, 0.02) | **0.17*****  **(0.10, 0.25)** | **0.12*****  **(0.06, 0.18)** |
| How happy have you felt during the last 7 days? | **0.26*****  **(0.18, 0.33)** | **0.12*****  **(0.06, 0.18)** | **0.14*****  **(0.06, 0.23)** | -0.03  (-0.10, 0.03) | **0.16*****  **(0.09, 0.24)** | **0.08***  **(0.02, 0.14)** |
| I expect more good things in my life than bad. | **0.28*****  **(0.20, 0.35)** | 0.05  (-0.01, 0.11) | **0.20*****  **(0.12, 0.28)** | 0.02  (-0.04, 0.08) | **0.10****  **(0.03, 0.18)** | 0.05  (-0.01, 0.11) |
| How would you rate your mental health? | **0.41*****  **(0.34, 0.48)** | 0.05  (-0.01, 0.10) | **0.14*****  **(0.06, 0.21)** | 0.01  (-0.05, 0.07) | **0.07***  **(0.00, 0.14)** | **0.07****  **(0.02, 0.13)** |
| Are you depressed? (reversed) | **0.51*****  **(0.44, 0.59)** | 0.03  (-0.03, 0.09) | 0.02  (-0.06, 0.10) | **-0.11*****  **(-0.17, -0.05)** | **0.10****  **(0.02, 0.17)** | **0.10*****  **(0.05, 0.16)** |
| Do you have anxiety that keeps you from doing the things in life that you need to do? (reversed) | **0.52*****  **(0.44, 0.60)** | 0.00  (-0.07, 0.06) | -0.06  (-0.15, 0.03) | -0.06  (-0.13, 0.01) | -0.04  (-0.12, 0.04) | 0.03  (-0.04, 0.09) |
| In stressful situations, I manage my emotions so that I am still in control of myself. | **0.38*****  **(0.30, 0.46)** | 0.03  (-0.04, 0.09) | 0.07  (-0.02, 0.15) | **0.24*****  **(0.17, 0.30)** | **-0.12****  **(-0.20, -0.05)** | 0.01  (-0.05, 0.08) |
|  |  |  |  |  |  |  |
| ***Physical Health*** |  |  |  |  |  |  |
| How would you rate your physical health? | 0.07  (-0.01, 0.14) | **0.43*****  **(0.37, 0.49)** | **0.10****  **(0.03, 0.18)** | -0.01  (-0.08, 0.05) | 0.01  (-0.07, 0.08) | **0.08****  **(0.02, 0.14)** |
| I have no major illnesses or injuries. | 0.01  (-0.07, 0.08) | **0.57*****  **(0.51, 0.63)** | -0.03  (-0.10, 0.05) | -0.04  (-0.10, 0.02) | 0.03  (-0.04, 0.10) | **0.08****  **(0.02, 0.14)** |
| I do not routinely get sick. | **0.10***  **(0.02, 0.18)** | **0.49*****  **(0.42, 0.55)** | 0.00  (-0.08, 0.09) | 0.01  (-0.06, 0.07) | -0.06  (-0.14, 0.02) | **0.08***  **(0.01, 0.14)** |
| My health does not prevent me from doing what I would like. | **0.09***  **(0.02, 0.16)** | **0.56*****  **(0.50, 0.62)** | 0.01  (-0.06, 0.09) | **-0.07***  **(-0.13, -0.01)** | 0.03  (-0.04, 0.10) | **0.08****  **(0.02, 0.13)** |
| My pain makes it hard for me to do my usual activities. (reversed) | **0.10***  **(0.02, 0.18)** | **0.50*****  **(0.43, 0.56)** | -0.02  (-0.11, 0.06) | **-0.07***  **(-0.14, 0.00)** | 0.04  (-0.04, 0.12) | -0.02  (-0.08, 0.05) |
| Based on my past health, I expect to be healthy long into the future. | 0.03  (-0.04, 0.09) | **0.63*****  **(0.58, 0.68)** | 0.04  (-0.03, 0.11) | -0.04  (-0.10, 0.02) | 0.05  (-0.02, 0.11) | 0.05  (0.00, 0.11) |
| I regularly do things to maintain and improve my health, in diet, exercise, and healthcare. | 0.03  (-0.05, 0.11) | **0.39*****  **(0.33, 0.45)** | 0.05  (-0.03, 0.13) | 0.01  (-0.06, 0.07) | 0.04  (-0.04, 0.11) | **0.09****  **(0.03, 0.16)** |
|  |  |  |  |  |  |  |
| ***Meaning and purpose*** |  |  |  |  |  |  |
| I have values and beliefs that help me understand who I am. | -0.03  (-0.10, 0.05) | 0.06  (0.00, 0.12) | **0.50*****  **(0.42, 0.58)** | 0.05  (-0.01, 0.12) | 0.00  (-0.07, 0.08) | 0.03  (-0.03, 0.09) |
| I know what gives meaning to my life. | -0.05  (-0.12, 0.03) | 0.03  (-0.03, 0.09) | **0.62*****  **(0.55, 0.70)** | -0.05  (-0.11, 0.02) | 0.03  (-0.04, 0.10) | 0.01  (-0.04, 0.07) |
| My life has a clear sense of purpose. | -0.04  (-0.11, 0.03) | **0.06***  **(0.01, 0.11)** | **0.58*****  **(0.51, 0.66)** | 0.00  (-0.05, 0.06) | **0.09****  **(0.03, 0.16)** | 0.02  (-0.03, 0.08) |
| I understand my purpose in life. | -0.06  (-0.13, 0.01) | **0.06***  **(0.00, 0.11)** | **0.62*****  **(0.55, 0.70)** | -0.01  (-0.07, 0.05) | **0.07***  **(0.01, 0.14)** | 0.05  (-0.01, 0.10) |
| To what extent do you feel the things you do in your life are worthwhile? | **0.08***  **(0.00, 0.16)** | **0.11*****  **(0.05, 0.17)** | **0.40*****  **(0.32, 0.48)** | -0.06  (-0.13, 0.00) | **0.13*****  **(0.05, 0.20)** | 0.04  (-0.02, 0.10) |
| I am pursuing what is most important to me in my life. | 0.04  (-0.03, 0.12) | 0.01  (-0.05, 0.07) | **0.46*****  **(0.38, 0.54)** | -0.01  (-0.07, 0.06) | **0.13*****  **(0.06, 0.21)** | **0.07***  **(0.01, 0.13)** |
|  |  |  |  |  |  |  |
| ***Character Strengths*** |  |  |  |  |  |  |
| I always act to promote good in all circumstances, even in difficult and challenging situations. | 0.03  (-0.04, 0.11) | **0.10****  **(0.04, 0.16)** | **0.15*****  **(0.07, 0.23)** | **0.41*****  **(0.35, 0.48)** | -0.02  (-0.10, 0.05) | 0.03  (-0.03, 0.09) |
| I always know the right thing to do. | 0.01  (-0.08, 0.09) | 0.01  (-0.05, 0.08) | **0.15****  **(0.06, 0.24)** | **0.35*****  **(0.28, 0.42)** | -0.02  (-0.10, 0.06) | **0.08***  **(0.02, 0.15)** |
| I always treat everyone with kindness, fairness and respect. | 0.03  (-0.05, 0.12) | 0.03  (-0.03, 0.10) | 0.02  (-0.07, 0.10) | **0.48*****  **(0.41, 0.55)** | 0.02  (-0.06, 0.10) | 0.01  (-0.05, 0.08) |
| I am always able to give up some happiness now for greater happiness later. | 0.08  (0.00, 0.16) | **0.07***  **(0.01, 0.13)** | 0.07  (-0.02, 0.15) | **0.40*****  **(0.33, 0.47)** | -0.01  (-0.09, 0.07) | **0.07***  **(0.00, 0.13)** |
| I am willing to face difficulties in order to do what is right. | 0.07  (-0.01, 0.15) | **0.07***  **(0.01, 0.13)** | **0.09***  **(0.00, 0.17)** | **0.46*****  **(0.40, 0.53)** | -0.07  (-0.14, 0.01) | 0.02  (-0.04, 0.08) |
| I give up personal pleasures whenever it is possible to do some good instead. | **-0.08***  **(-0.16, 0.00)** | **0.13*****  **(0.07, 0.19)** | **0.09***  **(0.01, 0.17)** | **0.49*****  **(0.43, 0.56)** | -0.05  (-0.13, 0.02) | 0.03  (-0.03, 0.09) |
| I get to use my strengths to help others. | **-0.08***  **(-0.16, -0.01)** | 0.05  (-0.01, 0.11) | **0.21*****  **(0.12, 0.29)** | **0.33*****  **(0.26, 0.39)** | **0.09***  **(0.01, 0.16)** | 0.03  (-0.03, 0.09) |
|  |  |  |  |  |  |  |
| ***Social Connectedness*** |  |  |  |  |  |  |
| My relationships are as satisfying as I would want them to be. | 0.04  (-0.04, 0.11) | -0.01  (-0.07, 0.04) | **0.16*****  **(0.08, 0.23)** | 0.00  (-0.06, 0.06) | **0.45*****  **(0.38, 0.52)** | **0.07***  **(0.01, 0.13)** |
| There are people who really understand me. | 0.03  (-0.05, 0.10) | 0.02  (-0.04, 0.07) | **0.15*****  **(0.07, 0.23)** | 0.00  (-0.07, 0.06) | **0.45*****  **(0.38, 0.53)** | 0.03  (-0.03, 0.09) |
| How often do you feel lonely? (reversed) | **0.31*****  **(0.24, 0.39)** | 0.04  (-0.02, 0.10) | 0.03  (-0.05, 0.11) | **-0.13*****  **(-0.20, -0.07)** | **0.30*****  **(0.23, 0.38)** | 0.05  (-0.01, 0.11) |
| I am content with my friendships and relationships. | 0.00  (-0.07, 0.08) | 0.03  (-0.03, 0.09) | **0.12****  **(0.04, 0.20)** | -0.02  (-0.08, 0.04) | **0.53*****  **(0.45, 0.60)** | 0.03  (-0.03, 0.09) |
| I have enough people I feel comfortable asking for help at any time. | 0.01  (-0.07, 0.08) | 0.02  (-0.04, 0.08) | 0.07  (-0.01, 0.15) | -0.04  (-0.10, 0.02) | **0.53*****  **(0.46, 0.61)** | 0.06  (0.00, 0.12) |
| I feel connected to the broader community around me. | -0.03  (-0.10, 0.04) | 0.03  (-0.03, 0.09) | **0.11****  **(0.04, 0.19)** | 0.02  (-0.04, 0.08) | **0.47*****  **(0.40, 0.54)** | **0.07***  **(0.01, 0.12)** |
| People in my broader community trust and respect one another. | 0.00  (-0.07, 0.08) | 0.06  (0.00, 0.12) | 0.05  (-0.03, 0.13) | 0.04  (-0.03, 0.10) | **0.43*****  **(0.35, 0.50)** | 0.03  (-0.03, 0.09) |
|  |  |  |  |  |  |  |
| ***Financial Security*** |  |  |  |  |  |  |
| I am able to meet my normal monthly living expenses without any difficulty. | -0.01  (-0.07, 0.06) | 0.02  (-0.03, 0.07) | -0.01  (-0.08, 0.07) | 0.03  (-0.03, 0.08) | 0.06  (0.00, 0.13) | **0.64*****  **(0.58, 0.69)** |
| How often do you worry about food, housing, or health expenses? (reversed) | **0.11****  **(0.04, 0.18)** | 0.05  (-0.01, 0.10) | 0.00  (-0.07, 0.08) | **-0.15*****  **(-0.21, -0.10)** | 0.06  (-0.01, 0.13) | **0.58*****  **(0.52, 0.63)** |
| I have sufficient savings that I could cover six months of expenses. | -0.03  (-0.09, 0.03) | -0.04  (-0.09, 0.00) | -0.02  (-0.09, 0.04) | 0.01  (-0.04, 0.06) | 0.03  (-0.03, 0.09) | **0.73*****  **(0.68, 0.78)** |
| My financial circumstances give me freedom to pursue my goals. | -0.04  (-0.10, 0.02) | -0.01  (-0.06, 0.04) | 0.03  (-0.04, 0.09) | 0.00  (-0.05, 0.05) | **0.09****  **(0.03, 0.15)** | **0.70*****  **(0.65, 0.74)** |
| Given my age, I have done adequate financial planning for the future. | **-0.08***  **(-0.14, -0.02)** | 0.00  (-0.05, 0.05) | **0.13*****  **(0.06, 0.20)** | -0.03  (-0.08, 0.02) | 0.04  (-0.02, 0.10) | **0.68*****  **(0.63, 0.72)** |
| The amount of debt I have often overwhelms me. (reversed) | **0.07***  **(0.00, 0.13)** | **-0.06***  **(-0.11, -0.01)** | -0.05  (-0.12, 0.02) | -0.01  (-0.06, 0.05) | 0.01  (-0.05, 0.08) | **0.72*****  **(0.67, 0.77)** |
| Note: The analyses examined the association between domains of flourishing (all domains included simultaneously) and subsequent well-being on individual survey items of flourishing (each individual item was examined one at a time). All models adjusted for gender, age, race, marital status, voting, education, number of children, care for older persons, home ownership, religious service attendance, spirituality, community participation, volunteering, work hours, salary bands, work type, and the number of physician-diagnosed physical health problems. The domain of physical health in the flourishing index assessed self-rated health, whereas the covariate of physician-diagnosed illness was an objective indicator of health. Likewise, the domain of social connectedness in the flourishing index assessed perceived social relationships, whereas the covariates of social participation measured objective features of social networks.  ^⸹^ Participants with missing data on the dependent variable were excluded from the analyses. Multiple imputation was performed to impute missing data on the independent variables and covariates.  **p*<.05 before Bonferroni correction, ***p<.*01 before Bonferroni correction, ****p<.*05 after Bonferroni correction (the p value cutoff for Bonferroni correction is p=0.05/40 tests=0.001). | | | | | | |

| **Supplementary Table S8. The longitudinal associations between domains of flourishing (other than the domain under which the item was taken as the dependent variable) and subsequent well-being on individual survey items of flourishing, adjusting for all individual items of the domain under which the dependent variable was taken from (N=1,209**^⸹^**).** | | | | | | |
| --- | --- | --- | --- | --- | --- | --- |
| **2018 Flourishing domains** | | | | | | |
| **2019 Flourishing individual survey items** | Emotional  health | Physical  health | Meaning and purpose | Character strengths | Social  connectedness | Financial  security |
| ***Emotional health*** |  |  |  |  |  |  |
| How satisfied are you with life as a whole these days? | ------- | 0.05  (0.00, 0.11) | **0.13****  **(0.05, 0.21)** | -0.04  (-0.11, 0.02) | **0.15*****  **(0.08, 0.22)** | **0.11*****  **(0.06, 0.17)** |
| How happy have you felt during the last 7 days? | ------- | **0.11*****  **(0.05, 0.17)** | **0.11***  **(0.03, 0.19)** | -0.03  (-0.10, 0.04) | **0.14*****  **(0.06, 0.21)** | **0.07***  **(0.01, 0.13)** |
| I expect more good things in my life than bad. | ------- | 0.04  (-0.01, 0.10) | **0.14*****  **(0.06, 0.21)** | -0.01  (-0.07, 0.05) | **0.10****  **(0.03, 0.17)** | **0.06***  **(0.00, 0.11)** |
| How would you rate your mental health? | ------- | 0.05  (-0.01, 0.10) | **0.09***  **(0.02, 0.17)** | -0.01  (-0.07, 0.05) | 0.06  (-0.01, 0.12) | **0.08****  **(0.02, 0.13)** |
| Are you depressed? (reversed) | ------- | 0.03  (-0.03, 0.09) | 0.04  (-0.04, 0.11) | **-0.11****  **(-0.17, -0.04)** | 0.06  (-0.01, 0.13) | **0.11*****  **(0.05, 0.16)** |
| Do you have anxiety that keeps you from doing the things in life that you need to do? (reversed) | ------- | 0.02  (-0.04, 0.08) | -0.01  (-0.10, 0.08) | **-0.09***  **(-0.16, -0.01)** | 0.01  (-0.07, 0.09) | 0.04  (-0.02, 0.10) |
| In stressful situations, I manage my emotions so that I am still in control of myself. | ------- | 0.03  (-0.02, 0.09) | 0.04  (-0.04, 0.12) | **0.12*****  **(0.05, 0.18)** | -0.07  (-0.14, 0.01) | 0.03  (-0.03, 0.09) |
|  |  |  |  |  |  |  |
| ***Physical Health*** |  |  |  |  |  |  |
| How would you rate your physical health? | 0.03  (-0.04, 0.11) | ------- | **0.09***  **(0.01, 0.16)** | -0.04  (-0.10, 0.02) | 0.00  (-0.07, 0.07) | **0.07***  **(0.01, 0.12)** |
| I have no major illnesses or injuries. | 0.02  (-0.05, 0.09) | ------- | -0.01  (-0.08, 0.07) | -0.03  (-0.09, 0.03) | 0.04  (-0.03, 0.11) | **0.07***  **(0.01, 0.12)** |
| I do not routinely get sick. | **0.11****  **(0.03, 0.19)** | ------- | 0.01  (-0.07, 0.09) | -0.03  (-0.09, 0.04) | -0.02  (-0.10, 0.05) | **0.08****  **(0.02, 0.14)** |
| My health does not prevent me from doing what I would like. | **0.10***  **(0.02, 0.17)** | ------- | 0.02  (-0.06, 0.10) | **-0.07***  **(-0.13, 0.00)** | 0.03  (-0.04, 0.10) | **0.09****  **(0.03, 0.15)** |
| My pain makes it hard for me to do my usual activities. (reversed) | **0.09***  **(0.01, 0.17)** | ------- | -0.02  (-0.10, 0.06) | -0.04  (-0.11, 0.02) | 0.02  (-0.06, 0.09) | 0.03  (-0.03, 0.09) |
| Based on my past health, I expect to be healthy long into the future. | 0.04  (-0.03, 0.11) | ------- | 0.02  (-0.05, 0.09) | -0.05  (-0.10, 0.01) | 0.04  (-0.03, 0.10) | 0.05  (0.00, 0.10) |
| I regularly do things to maintain and improve my health, in diet, exercise, and healthcare. | 0.04  (-0.03, 0.12) | ------- | 0.01  (-0.07, 0.08) | -0.03  (-0.09, 0.03) | 0.03  (-0.04, 0.10) | **0.08****  **(0.02, 0.13)** |
|  |  |  |  |  |  |  |
| ***Meaning and purpose*** |  |  |  |  |  |  |
| I have values and beliefs that help me understand who I am. | -0.01  (-0.09, 0.07) | 0.04  (-0.02, 0.10) | ------- | 0.03  (-0.03, 0.10) | 0.02  (-0.05, 0.09) | 0.05  (-0.01, 0.10) |
| I know what gives meaning to my life. | -0.02  (-0.10, 0.05) | 0.04  (-0.02, 0.09) | ------- | -0.03  (-0.10, 0.03) | 0.04  (-0.03, 0.12) | 0.02  (-0.03, 0.08) |
| My life has a clear sense of purpose. | -0.01  (-0.08, 0.06) | **0.07***  **(0.02, 0.12)** | ------- | 0.02  (-0.04, 0.08) | **0.09****  **(0.02, 0.16)** | 0.02  (-0.04, 0.07) |
| I understand my purpose in life. | -0.02  (-0.09, 0.05) | **0.07***  **(0.01, 0.12)** | ------- | 0.00  (-0.05, 0.06) | **0.07***  **(0.01, 0.14)** | 0.04  (-0.02, 0.09) |
| To what extent do you feel the things you do in your life are worthwhile? | 0.03  (-0.05, 0.11) | **0.11*****  **(0.05, 0.16)** | ------- | -0.04  (-0.11, 0.02) | **0.13*****  **(0.06, 0.21)** | 0.03  (-0.03, 0.09) |
| I am pursuing what is most important to me in my life. | 0.05  (-0.02, 0.13) | 0.02  (-0.03, 0.08) | ------- | 0.01  (-0.06, 0.07) | **0.12****  **(0.05, 0.19)** | 0.05  (0.00, 0.11) |
|  |  |  |  |  |  |  |
| ***Character Strengths*** |  |  |  |  |  |  |
| I always act to promote good in all circumstances, even in difficult and challenging situations. | -0.01  (-0.09, 0.07) | **0.10****  **(0.04, 0.15)** | **0.15*****  **(0.07, 0.23)** | ------- | -0.01  (-0.09, 0.06) | **0.06***  **(0.00, 0.12)** |
| I always know the right thing to do. | 0.00  (-0.09, 0.08) | 0.02  (-0.04, 0.08) | **0.16*****  **(0.08, 0.25)** | ------- | -0.04  (-0.12, 0.04) | **0.08***  **(0.01, 0.14)** |
| I always treat everyone with kindness, fairness and respect. | 0.02  (-0.06, 0.10) | 0.03  (-0.03, 0.09) | 0.06  (-0.03, 0.14) | ------- | 0.03  (-0.05, 0.10) | 0.04  (-0.02, 0.10) |
| I am always able to give up some happiness now for greater happiness later. | 0.07  (-0.01, 0.15) | **0.07***  **(0.00, 0.13)** | **0.09***  **(0.01, 0.18)** | ------- | 0.00  (-0.08, 0.08) | 0.06  (0.00, 0.12) |
| I am willing to face difficulties in order to do what is right. | 0.04  (-0.04, 0.12) | **0.07***  **(0.01, 0.13)** | **0.11***  **(0.02, 0.19)** | ------- | -0.05  (-0.13, 0.02) | 0.03  (-0.03, 0.09) |
| I give up personal pleasures whenever it is possible to do some good instead. | -0.07  (-0.15, 0.01) | **0.13*****  **(0.07, 0.19)** | **0.12****  **(0.03, 0.20)** | ------- | -0.04  (-0.11, 0.04) | 0.03  (-0.03, 0.09) |
| I get to use my strengths to help others. | -0.06  (-0.14, 0.02) | 0.05  (-0.01, 0.11) | **0.16*****  **(0.08, 0.25)** | ------- | 0.07  (0.00, 0.15) | 0.03  (-0.03, 0.09) |
|  |  |  |  |  |  |  |
| ***Social Connectedness*** |  |  |  |  |  |  |
| My relationships are as satisfying as I would want them to be. | 0.05  (-0.03, 0.12) | -0.01  (-0.06, 0.05) | **0.15*****  **(0.08, 0.23)** | -0.02  (-0.08, 0.04) | ------- | **0.07***  **(0.02, 0.13)** |
| There are people who really understand me. | 0.07  (-0.01, 0.15) | 0.02  (-0.03, 0.08) | **0.12****  **(0.04, 0.20)** | -0.01  (-0.07, 0.05) | ------- | 0.03  (-0.03, 0.09) |
| How often do you feel lonely? (reversed) | **0.19*****  **(0.11, 0.27)** | 0.03  (-0.03, 0.09) | 0.05  (-0.02, 0.13) | **-0.08***  **(-0.14, -0.02)** | ------- | **0.08****  **(0.02, 0.14)** |
| I am content with my friendships and relationships. | 0.01  (-0.07, 0.09) | 0.04  (-0.02, 0.09) | **0.12****  **(0.05, 0.20)** | -0.03  (-0.09, 0.04) | ------- | 0.05  (-0.01, 0.10) |
| I have enough people I feel comfortable asking for help at any time. | 0.07  (-0.01, 0.14) | 0.02  (-0.03, 0.08) | 0.06  (-0.02, 0.14) | -0.03  (-0.09, 0.03) | ------- | 0.05  (0.00, 0.11) |
| I feel connected to the broader community around me. | 0.01  (-0.06, 0.09) | 0.03  (-0.03, 0.08) | **0.11****  **(0.03, 0.19)** | 0.00  (-0.06, 0.06) | ------- | 0.06  (0.00, 0.11) |
| People in my broader community trust and respect one another. | 0.05  (-0.03, 0.13) | **0.06***  **(0.00, 0.12)** | 0.07  (-0.01, 0.15) | 0.00  (-0.06, 0.07) | ------- | 0.01  (-0.05, 0.06) |
|  |  |  |  |  |  |  |
| ***Financial Security*** |  |  |  |  |  |  |
| I am able to meet my normal monthly living expenses without any difficulty. | -0.02  (-0.08, 0.05) | 0.01  (-0.04, 0.06) | 0.02  (-0.05, 0.09) | 0.00  (-0.05, 0.06) | 0.05  (-0.01, 0.11) | ------- |
| How often do you worry about food, housing, or health expenses? (reversed) | **0.08***  **(0.01, 0.15)** | 0.03  (-0.03, 0.08) | 0.01  (-0.06, 0.09) | **-0.14*****  **(-0.20, -0.08)** | 0.05  (-0.01, 0.12) | ------- |
| I have sufficient savings that I could cover six months of expenses. | 0.00  (-0.05, 0.06) | -0.03  (-0.08, 0.01) | -0.01  (-0.07, 0.06) | 0.01  (-0.04, 0.06) | 0.04  (-0.02, 0.09) | ------- |
| My financial circumstances give me freedom to pursue my goals. | -0.03  (-0.09, 0.03) | -0.01  (-0.05, 0.04) | 0.02  (-0.05, 0.08) | -0.01  (-0.06, 0.04) | **0.09****  **(0.03, 0.15)** | ------- |
| Given my age, I have done adequate financial planning for the future. | -0.05  (-0.11, 0.01) | -0.02  (-0.06, 0.03) | **0.10****  **(0.04, 0.17)** | -0.04  (-0.09, 0.01) | 0.05  (0.00, 0.11) | ------- |
| The amount of debt I have often overwhelms me. (reversed) | 0.05  (-0.02, 0.11) | -0.05  (-0.09, 0.00) | -0.02  (-0.09, 0.05) | -0.01  (-0.06, 0.05) | 0.01  (-0.05, 0.07) | ------- |
| Note: The analyses examined the association between domains of flourishing (other than the domain from which the item was taken as the dependent variable, these domains were included simultaneously) and subsequent well-being on individual survey items of flourishing (each individual item was examined one at a time), adjusting for all individual items of the domain under which the dependent variable was taken from. All models adjusted for gender, age, race, marital status, voting, education, number of children, care for older persons, home ownership, religious service attendance, spirituality, community participation, volunteering, work hours, salary bands, work type, and the number of physician-diagnosed physical health problems. The domain of physical health in the flourishing index assessed self-rated health, whereas the covariate of physician-diagnosed illness was an objective indicator of health. Likewise, the domain of social connectedness in the flourishing index assessed perceived social relationships, whereas the covariates of social participation measured objective features of social networks.  ^⸹^ Participants with missing data on the dependent variable were excluded from the analyses. Multiple imputation was performed to impute missing data on the independent variables and covariates.  **p*<.05 before Bonferroni correction, ***p<.*01 before Bonferroni correction, ****p<.*05 after Bonferroni correction (the p value cutoff for Bonferroni correction is p=0.05/40 tests=0.001). | | | | | | |

| **Supplementary Table S9A. Individual survey items of the emotional health domain (examined one at a time) and subsequent flourishing (N=1,209**^⸹^**).** | | | | | | | | | |
| --- | --- | --- | --- | --- | --- | --- | --- | --- | --- |
| **2019 Flourishing** | | | | | | | | | |
| **2018 emotional**  **health items** | Emotional  health | Physical health | Meaning  and purpose | Character strengths | Social  connectedness | Financial security |  | Composite  flourishing | Composite flourishing (alternative)± |
| How satisfied are you with life as a whole these days | **0.16*****  **(0.10, 0.22)** | 0.04  (-0.01, 0.09) | -0.02  (-0.07, 0.04) | -0.01  (-0.07, 0.04) | **0.07***  **(0.01, 0.12)** | -0.03  (-0.07, 0.01) |  | 0.04  (-0.01, 0.09) | 0.01  (-0.04, 0.06) |
| How happy have you felt during the last 7 days? | **0.16*****  **(0.10, 0.22)** | **0.06***  **(0.01, 0.11)** | -0.05  (-0.10, 0.01) | 0.00  (-0.06, 0.05) | 0.02  (-0.04, 0.07) | -0.02  (-0.06, 0.02) |  | 0.03  (-0.02, 0.08) | 0.00  (-0.05, 0.05) |
| I expect more good things in my life than bad. | **0.23*****  **(0.17, 0.29)** | 0.01  (-0.04, 0.06) | **0.06***  **(0.00, 0.11)** | 0.03  (-0.03, 0.09) | 0.05  (-0.01, 0.10) | 0.00  (-0.05, 0.04) |  | **0.07****  **(0.02, 0.12)** | 0.03  (-0.01, 0.08) |
| How would you rate your mental health? | **0.35*****  **(0.29, 0.41)** | **0.06***  **(0.00, 0.12)** | -0.01  (-0.07, 0.05) | 0.00  (-0.07, 0.06) | 0.04  (-0.02, 0.10) | 0.02  (-0.03, 0.06) |  | **0.09*****  **(0.04, 0.14)** | 0.03  (-0.02, 0.08) |
| Are you depressed (reversed) | **0.30*****  **(0.24, 0.35)** | 0.02  (-0.03, 0.07) | 0.01  (-0.05, 0.06) | -0.01  (-0.07, 0.04) | 0.04  (-0.01, 0.09) | -0.01  (-0.05, 0.03) |  | **0.07****  **(0.02, 0.11)** | 0.01  (-0.03, 0.05) |
| Do you have anxiety that keeps you from doing the things in life that you need to do (reversed)? | **0.20*****  **(0.16, 0.25)** | 0.04  (-0.01, 0.08) | -0.02  (-0.07, 0.02) | 0.00  (-0.05, 0.05) | 0.00  (-0.04, 0.05) | 0.01  (-0.03, 0.04) |  | **0.04***  **(0.01, 0.08)** | 0.01  (-0.03, 0.05) |
| In stressful situations, I manage my emotions so that I am still in control of myself | **0.25*****  **(0.20, 0.30)** | 0.03  (-0.02, 0.08) | 0.02  (-0.04, 0.07) | 0.03  (-0.03, 0.08) | 0.04  (-0.01, 0.09) | 0.04  (0.00, 0.08) |  | **0.08*****  **(0.04, 0.13)** | 0.04  (0.00, 0.09) |
| Note: All models adjusted for gender, age, race, marital status, voting, education, number of children, care for older persons, home ownership, religious service attendance, spirituality, volunteering, community participation, work hours, salary bands, work type, number of diagnosed health problems, and all other 2018 flourishing domains (i.e., other than emotional health). The physical health domain in the flourishing index assessed self-rated health, whereas the covariate of physician-diagnosed illness was an objective indicator of health. Likewise, the domain of social connectedness in the flourishing index assessed perceived social relationships, whereas the covariates of social participation measured objective features of social networks.  ^⸹^  Participants with missing data on the dependent variable were excluded from the analyses. Multiple imputation was performed to impute missing data on the independent variables and covariates.  ± An alternative composite flourishing score excluding the specific domain under which the item was taken as the independent variable in each model.  **p*<.05 before Bonferroni correction, ***p<.*01 before Bonferroni correction, ****p<.*05 after Bonferroni correction (the p value cutoff for Bonferroni correction is p=0.05/56 tests=0.001). | | | | | | | | | |

| **Supplementary Table S9B. Individual items of the emotional health domain (examined simultaneously) and subsequent flourishing (N=1,209**^⸹^**).** | | | | | | | | | |
| --- | --- | --- | --- | --- | --- | --- | --- | --- | --- |
| **2019 Flourishing** | | | | | | | | | |
| **2018 emotional**  **health items** | Emotional  health | Physical health | Meaning  and purpose | Character strengths | Social  connectedness | Financial security |  | Composite  flourishing | Composite flourishing (alternative)± |
| How satisfied are you with life as a whole these days | 0.04  (-0.03, 0.10) | 0.01  (-0.05, 0.07) | 0.00  (-0.07, 0.06) | -0.02  (-0.09, 0.05) | **0.07***  **(0.00, 0.13)** | -0.03  (-0.08, 0.02) |  | 0.01  (-0.04, 0.06) | 0.00  (-0.05, 0.06) |
| How happy have you felt during the last 7 days? | -0.01  (-0.08, 0.05) | 0.04  (-0.02, 0.10) | -0.06  (-0.13, 0.00) | 0.00  (-0.06, 0.07) | -0.03  (-0.10, 0.03) | -0.01  (-0.06, 0.04) |  | -0.02  (-0.07, 0.04) | -0.02  (-0.07, 0.04) |
| I expect more good things in my life than bad. | **0.14*****  **(0.08, 0.19)** | -0.01  (-0.06, 0.05) | **0.07***  **(0.01, 0.13)** | 0.03  (-0.02, 0.09) | 0.03  (-0.03, 0.08) | 0.00  (-0.05, 0.04) |  | **0.05***  **(0.00, 0.10)** | 0.03  (-0.02, 0.08) |
| How would you rate your mental health? | **0.15*****  **(0.08, 0.22)** | 0.03  (-0.04, 0.10) | -0.01  (-0.08, 0.07) | -0.01  (-0.09, 0.07) | 0.01  (-0.07, 0.08) | 0.03  (-0.03, 0.09) |  | 0.04  (-0.02, 0.11) | 0.02  (-0.04, 0.08) |
| Are you depressed (reversed) | **0.16*****  **(0.10, 0.22)** | -0.01  (-0.07, 0.04) | 0.02  (-0.04, 0.08) | -0.02  (-0.08, 0.04) | 0.03  (-0.03, 0.09) | -0.02  (-0.07, 0.02) |  | 0.03  (-0.02, 0.08) | 0.00  (-0.05, 0.05) |
| Do you have anxiety that keeps you from doing the things in life that you need to do (reversed)? | **0.08*****  **(0.03, 0.13)** | 0.03  (-0.02, 0.07) | -0.03  (-0.08, 0.02) | 0.00  (-0.05, 0.05) | -0.02  (-0.07, 0.03) | 0.01  (-0.03, 0.04) |  | 0.01  (-0.03, 0.05) | 0.00  (-0.04, 0.04) |
| In stressful situations, I manage my emotions so that I am still in control of myself | **0.15*****  **(0.10, 0.21)** | 0.01  (-0.04, 0.06) | 0.02  (-0.03, 0.08) | 0.03  (-0.03, 0.09) | 0.04  (-0.02, 0.09) | 0.04  (0.00, 0.08) |  | **0.06****  **(0.01, 0.11)** | 0.04  (-0.01, 0.08) |
| Note: All models adjusted for gender, age, race, marital status, voting, education, number of children, care for older persons, home ownership, religious service attendance, spirituality, volunteering, community participation, work hours, salary bands, work type, number of diagnosed health problems, and all other 2018 flourishing domains (i.e., other than emotional health). The physical health domain in the flourishing index assessed self-rated health, whereas the covariate of physician-diagnosed illness was an objective indicator of health. Likewise, the domain of social connectedness in the flourishing index assessed perceived social relationships, whereas the covariates of social participation measured objective features of social networks.  ^⸹^  Participants with missing data on the dependent variable were excluded from the analyses. Multiple imputation was performed to impute missing data on the independent variables and covariates.  ± An alternative composite flourishing score excluding the specific domain under which the items were taken as the independent variables in each model.  **p*<.05 before Bonferroni correction, ***p<.*01 before Bonferroni correction, ****p<.*05 after Bonferroni correction (the p value cutoff for Bonferroni correction is p=0.05/8 tests=0.006). | | | | | | | | | |

| **Supplementary Table S10A. Individual items of the physical health domain (examined one at a time) and subsequent flourishing (N=1,209**^⸹^**).** | | | | | | | | | |
| --- | --- | --- | --- | --- | --- | --- | --- | --- | --- |
| **2019 Flourishing** | | | | | | | | | |
| **2018 physical health items** | Emotional  health | Physical health | Meaning  and purpose | Character strengths | Social  connectedness | Financial security |  | Composite  flourishing | Composite flourishing (alternative)± |
| How would you rate your physical health | -0.03  (-0.07, 0.02) | **0.33*****  **(0.27, 0.38)** | -0.01  (-0.06, 0.04) | 0.00  (-0.05, 0.05) | -0.02  (-0.07, 0.03) | -0.01  (-0.04, 0.03) |  | **0.06***  **(0.01, 0.10)** | -0.02  (-0.06, 0.03) |
| I have no major illnesses or injuries | **0.05***  **(0.00, 0.09)** | **0.39*****  **(0.34, 0.44)** | **0.06****  **(0.02, 0.11)** | **0.07****  **(0.02, 0.12)** | 0.03  (-0.01, 0.08) | -0.02  (-0.05, 0.02) |  | **0.11*****  **(0.08, 0.15)** | 0.04  (0.00, 0.08) |
| I do not routinely get sick | 0.04  (-0.01, 0.08) | **0.39*****  **(0.34, 0.44)** | 0.03  (-0.01, 0.08) | **0.06***  **(0.01, 0.11)** | 0.02  (-0.02, 0.07) | -0.01  (-0.04, 0.03) |  | **0.11*****  **(0.07, 0.14)** | 0.03  (-0.01, 0.07) |
| My health does not prevent me from doing what I would like | 0.04  (0.00, 0.09) | **0.46*****  **(0.41, 0.50)** | **0.05***  **(0.00, 0.09)** | **0.06***  **(0.01, 0.11)** | 0.04  (-0.01, 0.09) | -0.02  (-0.05, 0.02) |  | **0.12*****  **(0.08, 0.16)** | 0.03  (-0.01, 0.07) |
| My pain makes it hard for me to do my usual activities (reversed) | 0.01  (-0.03, 0.06) | **0.35*****  **(0.30, 0.40)** | 0.01  (-0.03, 0.06) | 0.02  (-0.03, 0.07) | 0.01  (-0.03, 0.06) | 0.00  (-0.04, 0.03) |  | **0.08*****  **(0.04, 0.12)** | 0.01  (-0.03, 0.05) |
| Based on my past health, I expect to be healthy long into the future | **0.08****  **(0.03, 0.12)** | **0.51*****  **(0.46, 0.56)** | **0.08****  **(0.03, 0.12)** | **0.11*****  **(0.06, 0.16)** | 0.03  (-0.02, 0.08) | -0.01  (-0.04, 0.03) |  | **0.16*****  **(0.12, 0.20)** | **0.06****  **(0.02, 0.10)** |
| I regularly do things to maintain and improve my health, in diet, exercise, and healthcare | 0.04  (-0.01, 0.08) | **0.33*****  **(0.27, 0.38)** | 0.02  (-0.03, 0.07) | 0.03  (-0.02, 0.08) | 0.00  (-0.04, 0.05) | 0.00  (-0.04, 0.04) |  | **0.08*****  **(0.04, 0.13)** | 0.02  (-0.02, 0.06) |
| Note: All models adjusted for gender, age, race, marital status, voting, education, number of children, care for older persons, home ownership, religious service attendance, spirituality, community participation, volunteering, work hours, salary bands, work type, number of diagnosed health problems, and all other 2018 flourishing domains (i.e., other than physical health). The physical health domain in the flourishing index assessed self-rated health, whereas the covariate of physician-diagnosed illness was an objective indicator of health. Likewise, the domain of social connectedness in the flourishing index assessed perceived social relationships, whereas the covariates of social participation measured objective features of social networks.  ^⸹^  Participants with missing data on the dependent variable were excluded from analyses. Multiple imputation was used to impute missing data on independent variables and covariates.  ± An alternative composite flourishing score excluding the specific domain under which the item was taken as the independent variable in each model.  **p*<.05 before Bonferroni correction, ***p<.*01 before Bonferroni correction, ****p<.*05 after Bonferroni correction (the p value cutoff for Bonferroni correction is p=0.05/56 tests=0.001). | | | | | | | | | |

| **Supplementary Table S10B. Individual items of the physical health domain (examined simultaneously) and subsequent flourishing (N=1,209**^⸹^**).** | | | | | | | | | |
| --- | --- | --- | --- | --- | --- | --- | --- | --- | --- |
| **2019 Flourishing** | | | | | | | | | |
| **2018 physical health items** | Emotional  health | Physical health | Meaning  and purpose | Character strengths | Social  connectedness | Financial security |  | Composite  flourishing | Composite flourishing (alternative)± |
| How would you rate your physical health | **-0.08****  **(-0.13, -0.02)** | **0.09*****  **(0.04, 0.14)** | -0.05  (-0.10, 0.01) | -0.05  (-0.11, 0.01) | -0.04  (-0.10, 0.01) | 0.00  (-0.05, 0.04) |  | -0.02  (-0.07, 0.03) | -0.05  (-0.10, 0.00) |
| I have no major illnesses or injuries | 0.02  (-0.04, 0.08) | **0.09*****  **(0.03, 0.14)** | 0.05  (-0.01, 0.11) | 0.02  (-0.04, 0.08) | 0.02  (-0.04, 0.07) | -0.01  (-0.06, 0.03) |  | 0.03  (-0.02, 0.08) | 0.02  (-0.03, 0.07) |
| I do not routinely get sick | 0.00  (-0.05, 0.05) | **0.15*****  **(0.10, 0.20)** | -0.01  (-0.07, 0.04) | 0.02  (-0.04, 0.07) | 0.00  (-0.05, 0.05) | 0.00  (-0.04, 0.04) |  | 0.03  (-0.01, 0.08) | 0.00  (-0.05, 0.05) |
| My health does not prevent me from doing what I would like | 0.01  (-0.06, 0.07) | **0.08***  **(0.01, 0.14)** | 0.01  (-0.06, 0.08) | 0.00  (-0.07, 0.07) | 0.04  (-0.03, 0.11) | -0.02  (-0.07, 0.03) |  | 0.02  (-0.04, 0.08) | 0.01  (-0.05, 0.06) |
| My pain makes it hard for me to do my usual activities (reversed) | -0.01  (-0.07, 0.04) | **0.15*****  **(0.10, 0.20)** | -0.02  (-0.07, 0.03) | -0.02  (-0.07, 0.04) | -0.01  (-0.06, 0.04) | 0.01  (-0.03, 0.05) |  | 0.02  (-0.02, 0.07) | -0.01  (-0.06, 0.04) |
| Based on my past health, I expect to be healthy long into the future | **0.08***  **(0.01, 0.14)** | **0.20*****  **(0.14, 0.27)** | **0.07***  **(0.00, 0.14)** | **0.12*****  **(0.05, 0.19)** | 0.01  (-0.05, 0.08) | 0.01  (-0.04, 0.06) |  | **0.09*****  **(0.04, 0.15)** | **0.06***  **(0.00, 0.12)** |
| I regularly do things to maintain and improve my health, in diet, exercise, and healthcare | 0.04  (-0.02, 0.09) | **0.11*****  **(0.06, 0.17)** | 0.01  (-0.05, 0.06) | 0.00  (-0.06, 0.06) | 0.00  (-0.05, 0.06) | 0.01  (-0.04, 0.05) |  | 0.03  (-0.01, 0.08) | 0.01  (-0.04, 0.06) |
| Note: All models adjusted for gender, age, race, marital status, voting, education, number of children, care for older persons, home ownership, religious service attendance, spirituality, community participation, volunteering, work hours, salary bands, work type, number of diagnosed health problems, and all other 2018 flourishing domains (i.e., other than physical health). The physical health domain in the flourishing index assessed self-rated health, whereas the covariate of physician-diagnosed illness was an objective indicator of health. Likewise, the domain of social connectedness in the flourishing index assessed perceived social relationships, whereas the covariates of social participation measured objective features of social networks.  ^⸹^  Participants with missing data on the dependent variable were excluded from analyses. Multiple imputation was used to impute missing data on independent variables and covariates.  ± An alternative composite flourishing score excluding the specific domain under which the items were taken as the independent variables in each model.  **p*<.05 before Bonferroni correction, ***p<.*01 before Bonferroni correction, ****p<.*05 after Bonferroni correction (the p value cutoff for Bonferroni correction is p=0.05/8 tests=0.006). | | | | | | | | | |

| **Supplementary Table S11A. Individual items of the meaning and purpose domain (examined one at a time) and subsequent flourishing (N=1,209**^⸹^**).** | | | | | | | | | |
| --- | --- | --- | --- | --- | --- | --- | --- | --- | --- |
| **2019 Flourishing** | | | | | | | | | |
| **2018 meaning and purpose items** | Emotional  health | Physical health | Meaning  and purpose | Character strengths | Social  connectedness | Financial security |  | Composite  flourishing | Composite flourishing (alternative)± |
| I have values and beliefs that help me understand who I am | 0.01  (-0.04, 0.07) | -0.04  (-0.09, 0.02) | **0.27*****  **(0.21, 0.33)** | 0.04  (-0.02, 0.10) | 0.03  (-0.02, 0.09) | 0.00  (-0.04, 0.04) |  | **0.06***  **(0.01, 0.11)** | 0.01  (-0.04, 0.06) |
| I know what gives meaning to my life | 0.04  (-0.02, 0.09) | -0.01  (-0.07, 0.04) | **0.36*****  **(0.30, 0.42)** | **0.12*****  **(0.06, 0.17)** | **0.06***  **(0.01, 0.11)** | 0.01  (-0.03, 0.06) |  | **0.11*****  **(0.06, 0.15)** | 0.05  (0.00, 0.09) |
| My life has a clear sense of purpose | **0.09****  **(0.03, 0.14)** | 0.05  (0.00, 0.11) | **0.44*****  **(0.38, 0.50)** | **0.12*****  **(0.06, 0.18)** | **0.11*****  **(0.05, 0.17)** | 0.03  (-0.02, 0.07) |  | **0.16*****  **(0.11, 0.21)** | **0.09*****  **(0.04, 0.14)** |
| I understand my purpose in life | **0.11*****  **(0.06, 0.17)** | 0.05  (-0.01, 0.10) | **0.45*****  **(0.39, 0.51)** | **0.12*****  **(0.06, 0.18)** | **0.11*****  **(0.05, 0.17)** | 0.02  (-0.02, 0.07) |  | **0.17*****  **(0.12, 0.22)** | **0.09*****  **(0.05, 0.14)** |
| to what extent do you feel the things you do in your life are worthwhile | **0.06***  **(0.01, 0.12)** | 0.03  (-0.02, 0.09) | **0.25*****  **(0.19, 0.31)** | **0.09****  **(0.03, 0.15)** | **0.08****  **(0.02, 0.14)** | -0.02  (-0.07, 0.02) |  | **0.09*****  **(0.04, 0.14)** | **0.05***  **(0.00, 0.10)** |
| I am pursuing what is most important to me in my life | 0.02  (-0.03, 0.08) | -0.01  (-0.06, 0.05) | **0.26*****  **(0.20, 0.32)** | 0.00  (-0.06, 0.06) | 0.01  (-0.05, 0.07) | 0.00  (-0.04, 0.04) |  | **0.06***  **(0.01, 0.10)** | 0.01  (-0.04, 0.05) |
| Note: All models adjusted for gender, age, race, marital status, voting, education, number of children, care for older persons, home ownership, religious service attendance, spirituality, community participation, volunteering, work hours, salary bands, work type, number of diagnosed health problems, and all other 2018 flourishing domains (i.e., other than purpose). The physical health domain in the flourishing index assessed self-rated health, whereas the covariate of physician-diagnosed illness was an objective indicator of health. Likewise, the domain of social connectedness in the flourishing index assessed perceived social relationships, whereas the covariates of social participation measured objective features of social networks.  ^⸹^  Participants with missing data on the dependent variable were excluded from analyses. Multiple imputation was used to impute missing data on independent variables and covariates.  ± An alternative composite flourishing score excluding the specific domain under which the item was taken as the independent variable in each model.  **p*<.05 before Bonferroni correction, ***p<.*01 before Bonferroni correction, ****p<.*05 after Bonferroni correction (the p value cutoff for Bonferroni correction is p=0.05/56 tests=0.001). | | | | | | | | | |

| **Supplementary Table S11B. Individual items of the meaning and purpose domain (examined simultaneously) and subsequent flourishing (N=1,209**^⸹^**).** | | | | | | | | | |
| --- | --- | --- | --- | --- | --- | --- | --- | --- | --- |
| **2019 Flourishing** | | | | | | | | | |
| **2018 meaning and purpose items** | Emotional  health | Physical health | Meaning  and purpose | Character strengths | Social  connectedness | Financial security |  | Composite  flourishing | Composite flourishing (alternative)± |
| I have values and beliefs that help me understand who I am | -0.04  (-0.11, 0.03) | **-0.07***  **(-0.13, -0.01)** | 0.04  (-0.02, 0.11) | -0.06  (-0.12, 0.01) | -0.04  (-0.10, 0.03) | -0.02  (-0.07, 0.04) |  | -0.04  (-0.09, 0.02) | -0.05  (-0.11, 0.00) |
| I know what gives meaning to my life | 0.00  (-0.07, 0.07) | -0.03  (-0.10, 0.03) | **0.15*****  **(0.08, 0.22)** | **0.10****  **(0.03, 0.17)** | 0.02  (-0.05, 0.09) | 0.01  (-0.04, 0.07) |  | 0.05  (-0.01, 0.11) | 0.02  (-0.04, 0.08) |
| My life has a clear sense of purpose | -0.01  (-0.11, 0.10) | 0.08  (-0.02, 0.19) | 0.10  (-0.01, 0.20) | 0.05  (-0.07, 0.16) | 0.06  (-0.05, 0.17) | 0.04  (-0.05, 0.12) |  | 0.07  (-0.03, 0.16) | 0.06  (-0.03, 0.15) |
| I understand my purpose in life | **0.13****  **(0.03, 0.23)** | 0.03  (-0.06, 0.13) | **0.23*****  **(0.13, 0.34)** | 0.07  (-0.04, 0.18) | 0.07  (-0.03, 0.17) | 0.00  (-0.08, 0.08) |  | **0.10***  **(0.02, 0.19)** | 0.07  (-0.02, 0.16) |
| To what extent do you feel the things you do in your life are worthwhile | 0.04  (-0.02, 0.10) | 0.03  (-0.03, 0.09) | **0.08****  **(0.02, 0.15)** | 0.05  (-0.01, 0.12) | 0.06  (0.00, 0.12) | -0.03  (-0.08, 0.01) |  | 0.04  (-0.01, 0.09) | 0.03  (-0.02, 0.08) |
| I am pursuing what is most important to me in my life | -0.02  (-0.09, 0.04) | -0.04  (-0.10, 0.02) | 0.06  (-0.01, 0.12) | **-0.08***  **(-0.15, -0.01)** | -0.06  (-0.12, 0.01) | -0.01  (-0.06, 0.04) |  | -0.03  (-0.08, 0.03) | -0.05  (-0.10, 0.01) |
| Note: All models adjusted for gender, age, race, marital status, voting, education, number of children, care for older persons, home ownership, religious service attendance, spirituality, community participation, volunteering, work hours, salary bands, work type, number of diagnosed health problems, and all other 2018 flourishing domains (i.e., other than purpose). The physical health domain in the flourishing index assessed self-rated health, whereas the covariate of physician-diagnosed illness was an objective indicator of health. Likewise, the domain of social connectedness in the flourishing index assessed perceived social relationships, whereas the covariates of social participation measured objective features of social networks.  ^⸹^  Participants with missing data on the dependent variable were excluded from analyses. Multiple imputation was used to impute missing data on independent variables and covariates.  ± An alternative composite flourishing score excluding the specific domain under which the items were taken as the independent variables in each model.  **p*<.05 before Bonferroni correction, ***p<.*01 before Bonferroni correction, ****p<.*05 after Bonferroni correction (the p value cutoff for Bonferroni correction is p=0.05/8 tests =0.006). | | | | | | | | | |

| **Supplementary Table S12A. Individual items of the character strength domain (examined one at a time) and subsequent flourishing (N=1,209**^⸹^**).** | | | | | | | | | |
| --- | --- | --- | --- | --- | --- | --- | --- | --- | --- |
| **2019 Flourishing** | | | | | | | | | |
| **2018 character strengths items** | Emotional  health | Physical health | Meaning  and purpose | Character strengths | Social  connectedness | Financial security |  | Composite  flourishing | Composite flourishing (alternative)± |
| I always act to promote good in all circumstances, even in difficult and challenging situations. | 0.04  (-0.01, 0.08) | -0.01  (-0.06, 0.03) | 0.01  (-0.04, 0.06) | **0.36*****  **(0.31, 0.42)** | 0.05  (0.00, 0.10) | 0.02  (-0.02, 0.06) |  | **0.08*****  **(0.03, 0.12)** | 0.03  (-0.02, 0.07) |
| I always know the right thing to do | -0.02  (-0.07, 0.03) | -0.04  (-0.08, 0.01) | -0.02  (-0.07, 0.03) | **0.22*****  **(0.17, 0.28)** | -0.04  (-0.09, 0.00) | -0.04  (-0.07, 0.00) |  | 0.00  (-0.04, 0.04) | **-0.04***  **(-0.08, 0.00)** |
| I always treat everyone with kindness, fairness and respect | 0.00  (-0.04, 0.04) | -0.02  (-0.07, 0.02) | 0.00  (-0.04, 0.05) | **0.34*****  **(0.29, 0.39)** | -0.01  (-0.06, 0.03) | **-0.04***  **(-0.07, 0.00)** |  | 0.03  (-0.01, 0.07) | -0.02  (-0.06, 0.02) |
| I am always able to give up some happiness now for greater happiness later. | -0.02  (-0.06, 0.03) | 0.00  (-0.04, 0.04) | -0.02  (-0.07, 0.02) | **0.29*****  **(0.23, 0.34)** | -0.03  (-0.08, 0.01) | 0.01  (-0.03, 0.04) |  | 0.03  (-0.01, 0.07) | -0.01  (-0.05, 0.02) |
| I am willing to face difficulties in order to do what is right | 0.00  (-0.05, 0.05) | -0.04  (-0.09, 0.00) | 0.01  (-0.04, 0.06) | **0.29*****  **(0.24, 0.34)** | -0.02  (-0.07, 0.03) | -0.03  (-0.07, 0.01) |  | 0.02  (-0.02, 0.06) | -0.02  (-0.06, 0.02) |
| I give up personal pleasures whenever it is possible to do some good instead | -0.01  (-0.06, 0.03) | -0.03  (-0.07, 0.02) | -0.03  (-0.07, 0.02) | **0.28*****  **(0.23, 0.33)** | -0.01  (-0.06, 0.03) | -0.01  (-0.04, 0.03) |  | 0.02  (-0.02, 0.06) | -0.02  (-0.06, 0.02) |
| I get to use my strengths to help others | -0.02  (-0.07, 0.03) | -0.02  (-0.07, 0.03) | 0.02  (-0.04, 0.07) | **0.21*****  **(0.15, 0.27)** | -0.03  (-0.08, 0.02) | -0.02  (-0.06, 0.02) |  | 0.01  (-0.03, 0.05) | -0.02  (-0.06, 0.02) |
| Note: All models adjusted for gender, age, race, marital status, voting, education, number of children, care for older persons, home ownership, religious service attendance, spirituality, community participation, volunteering, work hours, salary bands, work type, number of diagnosed health problems, and all other 2018 flourishing domains (i.e., other than character strengths). The physical health domain in the flourishing index assessed self-rated health, whereas the covariate of physician-diagnosed illness was an objective indicator of health. Likewise, the domain of social connectedness in the flourishing index assessed perceived social relationships, whereas the covariates of social participation measured objective features of social networks.  ^⸹^  Participants with missing data on the dependent variable were excluded from analyses. Multiple imputation was used to impute missing data on independent variables and covariates.  ± An alternative composite flourishing score excluding the specific domain under which the item was taken as the independent variable in each model.  *p<.05 before Bonferroni correction, **p<.01 before Bonferroni correction, ***p<.05 after Bonferroni correction (the p value cutoff for Bonferroni correction is p=0.05/56 tests=0.001). | | | | | | | | | |

| **Supplementary Table S12B. Individual items of the character strength domain (examined simultaneously) and subsequent flourishing (N=1,209**^⸹^**).** | | | | | | | | | |
| --- | --- | --- | --- | --- | --- | --- | --- | --- | --- |
| **2019 Flourishing** | | | | | | | | |  |
| **2018 character strengths items** | Emotional  health | Physical health | Meaning  and purpose | Character strengths | Social  connectedness | Financial security |  | Composite  flourishing | Composite flourishing (alternative)± |
| I always act to promote good in all circumstances, even in difficult and challenging situations. | **0.06***  **(0.00, 0.12)** | 0.02  (-0.04, 0.07) | 0.02  (-0.04, 0.08) | **0.15*****  **(0.09, 0.22)** | **0.09****  **(0.03, 0.15)** | **0.07*****  **(0.02, 0.12)** |  | **0.08*****  **(0.03, 0.14)** | **0.07****  **(0.02, 0.12)** |
| I always know the right thing to do | -0.03  (-0.08, 0.02) | -0.03  (-0.08, 0.02) | -0.02  (-0.07, 0.03) | **0.08*****  **(0.03, 0.14)** | **-0.05***  **(-0.10, 0.00)** | -0.04  (-0.08, 0.00) |  | -0.03  (-0.07, 0.02) | **-0.04***  **(-0.09, 0.00)** |
| I always treat everyone with kindness, fairness and respect | -0.01  (-0.06, 0.04) | -0.02  (-0.07, 0.04) | 0.00  (-0.05, 0.05) | **0.16*****  **(0.11, 0.22)** | -0.02  (-0.08, 0.03) | **-0.05***  **(-0.09, -0.01)** |  | 0.00  (-0.05, 0.04) | -0.03  (-0.08, 0.01) |
| I am always able to give up some happiness now for greater happiness later. | -0.02  (-0.08, 0.03) | 0.03  (-0.02, 0.09) | -0.03  (-0.08, 0.03) | **0.10*****  **(0.04, 0.15)** | -0.03  (-0.09, 0.02) | 0.03  (-0.01, 0.07) |  | 0.01  (-0.03, 0.06) | 0.00  (-0.05, 0.05) |
| I am willing to face difficulties in order to do what is right | 0.01  (-0.05, 0.07) | -0.04  (-0.10, 0.01) | 0.03  (-0.03, 0.09) | **0.07***  **(0.00, 0.13)** | -0.01  (-0.06, 0.05) | -0.04  (-0.08, 0.01) |  | -0.01  (-0.06, 0.04) | -0.02  (-0.07, 0.03) |
| I give up personal pleasures whenever it is possible to do some good instead | -0.01  (-0.06, 0.04) | -0.02  (-0.07, 0.04) | -0.04  (-0.10, 0.02) | **0.08****  **(0.03, 0.14)** | 0.01  (-0.05, 0.06) | 0.01  (-0.04, 0.05) |  | 0.00  (-0.05, 0.05) | -0.01  (-0.06, 0.04) |
| I get to use my strengths to help others | -0.02  (-0.07, 0.03) | -0.01  (-0.06, 0.04) | 0.03  (-0.03, 0.08) | 0.05  (-0.01, 0.11) | -0.03  (-0.08, 0.03) | -0.02  (-0.06, 0.03) |  | 0.00  (-0.05, 0.04) | -0.01  (-0.06, 0.03) |
| Note: All models adjusted for gender, age, race, marital status, voting, education, number of children, care for older persons, home ownership, religious service attendance, spirituality, community participation, volunteering, work hours, salary bands, work type, number of diagnosed health problems, and all other 2018 flourishing domains (i.e., other than character strengths). The physical health domain in the flourishing index assessed self-rated health, whereas the covariate of physician-diagnosed illness was an objective indicator of health. Likewise, the domain of social connectedness in the flourishing index assessed perceived social relationships, whereas the covariates of social participation measured objective features of social networks.  ^⸹^  Participants with missing data on the dependent variable were excluded from the analyses. Multiple imputation was performed to impute missing data on the independent variables and covariates.  ± An alternative composite flourishing score excluding the specific domain under which the items were taken as the independent variables in each model.  **p*<.05 before Bonferroni correction, ***p<.*01 before Bonferroni correction, ****p<.*05 after Bonferroni correction (the p value cutoff for Bonferroni correction is p=0.05/8 tests=0.006). | | | | | | | | | |

| **Supplementary Table S13A. Individual items of the social connectedness domain (examined one at a time) and subsequent flourishing (N=1,209**^⸹^**).** | | | | | | | | | |
| --- | --- | --- | --- | --- | --- | --- | --- | --- | --- |
| **2019 Flourishing** | | | | | | | | | |
| **2018 social connectedness items** | Emotional  health | Physical health | Meaning  and purpose | Character strengths | Social  connectedness | Financial security |  | Composite  flourishing | Composite flourishing (alternative)± |
| My relationships are as satisfying as I would want them to be | **0.06***  **(0.01, 0.11)** | **0.05***  **(0.00, 0.11)** | **0.06***  **(0.00, 0.11)** | -0.03  (-0.08, 0.03) | **0.32*****  **(0.27, 0.38)** | 0.03  (-0.02, 0.07) |  | **0.11*****  **(0.06, 0.16)** | **0.05***  **(0.00, 0.09)** |
| There are people who really understand me | 0.05  (0.00, 0.10) | 0.01  (-0.04, 0.06) | **0.07****  **(0.02, 0.12)** | 0.03  (-0.03, 0.08) | **0.30*****  **(0.24, 0.35)** | 0.04  (0.00, 0.08) |  | **0.11*****  **(0.06, 0.15)** | **0.05***  **(0.01, 0.09)** |
| How often do you feel lonely (reversed) | **0.06***  **(0.01, 0.12)** | -0.02  (-0.07, 0.03) | 0.03  (-0.02, 0.09) | 0.02  (-0.04, 0.07) | **0.19*****  **(0.13, 0.24)** | 0.00  (-0.05, 0.04) |  | **0.06***  **(0.01, 0.10)** | 0.02  (-0.03, 0.06) |
| I am content with my friendships and relationships | 0.03  (-0.02, 0.08) | 0.03  (-0.02, 0.08) | **0.07***  **(0.01, 0.12)** | 0.00  (-0.06, 0.05) | **0.38*****  **(0.33, 0.44)** | 0.03  (-0.02, 0.07) |  | **0.12*****  **(0.07, 0.16)** | 0.04  (0.00, 0.09) |
| I have enough people I feel comfortable asking for help at any time | 0.04  (-0.01, 0.09) | 0.02  (-0.02, 0.07) | 0.05  (0.00, 0.10) | 0.00  (-0.05, 0.05) | **0.33*****  **(0.28, 0.39)** | **0.06****  **(0.02, 0.10)** |  | **0.11*****  **(0.07, 0.16)** | **0.05***  **(0.01, 0.09)** |
| I feel connected to the broader community around me | 0.01  (-0.04, 0.06) | 0.02  (-0.03, 0.07) | 0.03  (-0.02, 0.09) | -0.04  (-0.09, 0.02) | **0.30*****  **(0.24, 0.35)** | 0.02  (-0.02, 0.06) |  | **0.08****  **(0.03, 0.12)** | 0.02  (-0.03, 0.06) |
| People in my broader community trust and respect one another. | 0.02  (-0.03, 0.07) | -0.02  (-0.06, 0.03) | 0.03  (-0.02, 0.08) | -0.03  (-0.08, 0.03) | **0.24*****  **(0.19, 0.29)** | 0.04  (0.00, 0.08) |  | **0.07****  **(0.02, 0.11)** | 0.02  (-0.02, 0.06) |
| Note: All models adjusted for gender, age, race, marital status, voting, education, number of children, care for older persons, home ownership, religious service attendance, spirituality, community participation, volunteering, work hours, salary bands, work type, number of diagnosed health problems, and all other 2018 flourishing domains (i.e., other than social connectedness). The physical health domain in the flourishing index assessed self-rated health, whereas the covariate of physician-diagnosed illness was an objective indicator of health. Likewise, the domain of social connectedness in the flourishing index assessed perceived social relationships, whereas the covariates of social participation measured objective features of social networks.  ^⸹^  Participants with missing data on the dependent variable were excluded from the analyses. Multiple imputation was performed to impute missing data on the independent variables and covariates.  ± An alternative composite flourishing score excluding the specific domain under which the item was taken as the independent variable in each model.  **p*<.05 before Bonferroni correction, ***p<.*01 before Bonferroni correction, ****p<.*05 after Bonferroni correction (the p value cutoff for Bonferroni correction is p=0.05/56 tests=0.001). | | | | | | | | | |

| **Supplementary Table S13B. Individual items of the social connectedness domain (examined simultaneously) and subsequent flourishing (N=1,209**^⸹^**).** | | | | | | | | | |
| --- | --- | --- | --- | --- | --- | --- | --- | --- | --- |
| **2019 Flourishing** | | | | | | | | | |
| **2018 social connectedness items** | Emotional  health | Physical health | Meaning  and purpose | Character strengths | Social  connectedness | Financial security |  | Composite  flourishing | Composite flourishing (alternative)± |
| My relationships are as satisfying as I would want them to be | 0.06  (-0.01, 0.13) | 0.06  (-0.01, 0.13) | 0.02  (-0.06, 0.09) | -0.04  (-0.12, 0.03) | **0.08***  **(0.01, 0.16)** | 0.01  (-0.04, 0.07) |  | 0.04  (-0.02, 0.10) | 0.03  (-0.03, 0.09) |
| There are people who really understand me | 0.03  (-0.03, 0.09) | -0.02  (-0.08, 0.04) | 0.05  (-0.02, 0.11) | 0.04  (-0.02, 0.11) | **0.08****  **(0.02, 0.15)** | 0.02  (-0.03, 0.07) |  | 0.04  (-0.01, 0.10) | 0.03  (-0.02, 0.08) |
| How often do you feel lonely (reversed) | 0.05  (0.00, 0.11) | -0.03  (-0.09, 0.02) | 0.01  (-0.05, 0.07) | 0.02  (-0.04, 0.08) | **0.07***  **(0.02, 0.13)** | -0.02  (-0.06, 0.02) |  | 0.02  (-0.03, 0.07) | 0.00  (-0.04, 0.05) |
| I am content with my friendships and relationships | -0.04  (-0.12, 0.04) | 0.00  (-0.07, 0.08) | 0.02  (-0.06, 0.10) | 0.01  (-0.08, 0.09) | **0.14*****  **(0.06, 0.22)** | -0.03  (-0.09, 0.04) |  | 0.02  (-0.05, 0.09) | -0.01  (-0.08, 0.06) |
| I have enough people I feel comfortable asking for help at any time | 0.02  (-0.04, 0.08) | 0.02  (-0.04, 0.08) | 0.01  (-0.05, 0.07) | 0.01  (-0.06, 0.07) | **0.13*****  **(0.07, 0.20)** | **0.06***  **(0.01, 0.11)** |  | **0.06***  **(0.01, 0.11)** | 0.04  (-0.02, 0.09) |
| I feel connected to the broader community around me | -0.03  (-0.10, 0.04) | 0.04  (-0.03, 0.11) | -0.01  (-0.09, 0.07) | -0.04  (-0.12, 0.04) | 0.07  (-0.01, 0.14) | -0.04  (-0.10, 0.02) |  | 0.00  (-0.07, 0.06) | -0.02  (-0.08, 0.04) |
| People in my broader community trust and respect one another. | 0.02  (-0.05, 0.08) | -0.05  (-0.12, 0.01) | 0.02  (-0.05, 0.08) | -0.01  (-0.08, 0.06) | **0.08***  **(0.02, 0.15)** | 0.04  (-0.01, 0.09) |  | 0.03  (-0.03, 0.08) | 0.01  (-0.05, 0.07) |
| Note: All models adjusted for gender, age, race, marital status, voting, education, number of children, care for older persons, home ownership, religious service attendance, spirituality, community participation, volunteering, work hours, salary bands, work type, number of diagnosed health problems, and all other 2018 flourishing domains (i.e., other than social connectedness). The physical health domain in the flourishing index assessed self-rated health, whereas the covariate of physician-diagnosed illness was an objective indicator of health. Likewise, the domain of social connectedness in the flourishing index assessed perceived social relationships, whereas the covariates of social participation measured objective features of social networks.  ^⸹^  Participants with missing data on the dependent variable were excluded from the analyses. Multiple imputation was performed to impute missing data on the independent variables and covariates.  ± An alternative composite flourishing score excluding the specific domain under which the items were taken as the independent variables in each model.  **p*<.05 before Bonferroni correction, ***p<.*01 before Bonferroni correction, ****p<.*05 after Bonferroni correction (the p value cutoff for Bonferroni correction is p=0.05/8 tests=0.006). | | | | | | | | | |

| **Supplementary Table S14A. Individual items of the financial security domain (examined one at a time) and subsequent flourishing (N=1,209**^⸹^**).** | | | | | | | | | |
| --- | --- | --- | --- | --- | --- | --- | --- | --- | --- |
| **2019 Flourishing** | | | | | | | | | |
| **2018 financial security items** | Emotional  health | Physical health | Meaning  and purpose | Character strengths | Social  connectedness | Financial security |  | Composite  flourishing | Composite flourishing (alternative)± |
| I am able to meet my normal monthly living expenses without any difficulty | **0.07****  **(0.03, 0.12)** | 0.03  (-0.02, 0.08) | **0.06****  **(0.02, 0.11)** | 0.04  (-0.01, 0.09) | **0.07****  **(0.02, 0.11)** | **0.58*****  **(0.54, 0.63)** |  | **0.25*****  **(0.20, 0.29)** | **0.07****  **(0.02, 0.11)** |
| How often do you worry about food, housing, or health expenses? | **0.06****  **(0.02, 0.11)** | 0.04  (-0.01, 0.08) | 0.02  (-0.03, 0.07) | 0.02  (-0.03, 0.08) | 0.04  (-0.01, 0.09) | **0.49*****  **(0.44, 0.54)** |  | **0.20*** (0.16, 0.24)** | **0.05***  **(0.00, 0.09)** |
| I have sufficient savings that I could cover six months of expenses | **0.06****  **(0.02, 0.11)** | **0.06****  **(0.02, 0.11)** | 0.02  (-0.02, 0.07) | **0.06***  **(0.01, 0.11)** | 0.04  (-0.01, 0.09) | **0.63*****  **(0.59, 0.67)** |  | **0.26*** (0.22, 0.30)** | **0.06****  **(0.02, 0.10)** |
| My financial circumstances give me freedom to pursue my goals | **0.06***  **(0.01, 0.11)** | **0.05***  **(0.01, 0.10)** | 0.03  (-0.02, 0.08) | 0.02  (-0.03, 0.07) | 0.04  (-0.01, 0.09) | **0.62*****  **(0.57, 0.66)** |  | **0.24*** (0.20, 0.29)** | **0.05***  **(0.00, 0.09)** |
| Given my age, I have done adequate financial planning for the future | **0.06***  **(0.01, 0.11)** | **0.09*****  **(0.05, 0.14)** | 0.03  (-0.02, 0.08) | 0.02  (-0.03, 0.07) | 0.03  (-0.01, 0.08) | **0.58*****  **(0.53, 0.62)** |  | **0.24*** (0.20, 0.28)** | **0.06****  **(0.01, 0.11)** |
| The amount of debt I have often overwhelms me. | **0.05***  **(0.01, 0.10)** | **0.06***  **(0.01, 0.10)** | 0.03  (-0.02, 0.07) | **0.05***  **(0.00, 0.10)** | 0.04  (-0.01, 0.09) | **0.52*****  **(0.47, 0.56)** |  | **0.21*** (0.17, 0.26)** | **0.05***  **(0.01, 0.10)** |
| Note: All models adjusted for gender, age, race, marital status, voting, education, number of children, care for older persons, home ownership, religious service attendance, spirituality, community participation, volunteering, work hours, salary bands, work type, number of diagnosed health problems, and all other 2018 flourishing domains (i.e., other than financial security). The physical health domain in the flourishing index assessed self-rated health, whereas the covariate of physician-diagnosed illness was an objective indicator of health. Likewise, the domain of social connectedness in the flourishing index assessed perceived social relationships, whereas the covariates of social participation measured objective features of social networks.  ^⸹^  Participants with missing data on the dependent variable were excluded from the analyses. Multiple imputation was performed to impute missing data on the independent variables and covariates.  ± An alternative composite flourishing score excluding the specific domain under which the item was taken as the independent variable in each model.  **p*<.05 before Bonferroni correction, ***p<.*01 before Bonferroni correction, ****p<.*05 after Bonferroni correction (the p value cutoff for Bonferroni correction is p=0.05/56 tests=0.001). | | | | | | | | | |

| **Supplementary Table S14B. Individual items of the financial security domain (examined simultaneously) and subsequent flourishing (N=1,209**^⸹^**).** | | | | | | | | | |
| --- | --- | --- | --- | --- | --- | --- | --- | --- | --- |
| **2019 Flourishing** | | | | | | | | | |
| **2018 financial security items** | Emotional  health | Physical health | Meaning  and purpose | Character strengths | Social  connectedness | Financial security |  | Composite  flourishing | Composite flourishing (alternative)± |
| I am able to meet my normal monthly living expenses without any difficulty. | 0.04  (-0.03, 0.11) | -0.03  (-0.09, 0.04) | **0.09***  **(0.02, 0.16)** | 0.02  (-0.05, 0.10) | 0.06  (-0.01, 0.13) | **0.17*****  **(0.12, 0.22)** |  | **0.09****  **(0.03, 0.15)** | 0.04  (-0.02, 0.11) |
| How often do you worry about food, housing, or health expenses? | 0.03  (-0.03, 0.09) | 0.00  (-0.06, 0.06) | -0.03  (-0.09, 0.04) | -0.01  (-0.07, 0.06) | 0.01  (-0.06, 0.07) | **0.12*****  **(0.07, 0.16)** |  | 0.04  (-0.01, 0.09) | 0.00  (-0.06, 0.06) |
| I have sufficient savings that I could cover six months of expenses. | 0.03  (-0.04, 0.11) | 0.02  (-0.05, 0.09) | -0.02  (-0.09, 0.06) | **0.10***  **(0.02, 0.18)** | 0.01  (-0.06, 0.09) | **0.27*****  **(0.21, 0.33)** |  | **0.11*****  **(0.05, 0.18)** | 0.03  (-0.04, 0.10) |
| My financial circumstances give me freedom to pursue my goals. | -0.02  (-0.11, 0.06) | -0.02  (-0.10, 0.06) | -0.02  (-0.11, 0.06) | -0.07  (-0.16, 0.02) | -0.02  (-0.10, 0.07) | **0.07***  **(0.00, 0.13)** |  | -0.01  (-0.08, 0.07) | -0.03  (-0.11, 0.04) |
| Given my age, I have done adequate financial planning for the future. | 0.02  (-0.05, 0.09) | **0.09****  **(0.03, 0.16)** | 0.02  (-0.05, 0.10) | -0.02  (-0.10, 0.05) | 0.00  (-0.07, 0.07) | **0.16*****  **(0.10, 0.21)** |  | **0.08***  **(0.02, 0.14)** | 0.03  (-0.03, 0.10) |
| The amount of debt I have often overwhelms me. | 0.00  (-0.06, 0.06) | 0.03  (-0.02, 0.09) | 0.00  (-0.06, 0.07) | 0.04  (-0.02, 0.10) | 0.01  (-0.05, 0.07) | **0.14*****  **(0.09, 0.19)** |  | **0.06***  **(0.01, 0.11)** | 0.02  (-0.04, 0.08) |
| Note: All models adjusted for gender, age, race, marital status, voting, education, number of children, care for older persons, home ownership, religious service attendance, spirituality, community participation, volunteering, work hours, salary bands, work type, number of diagnosed health problems, and all other 2018 flourishing domains (i.e., other than financial security). The physical health domain in the flourishing index assessed self-rated health, whereas the covariate of physician-diagnosed illness was an objective indicator of health. Likewise, the domain of social connectedness in the flourishing index assessed perceived social relationships, whereas the covariates of social participation measured objective features of social networks.  ^⸹^  Participants with missing data on the dependent variable were excluded from the analyses. Multiple imputation was used to impute missing data on the independent variables and covariates.  ± An alternative composite flourishing score excluding the specific domain under which the items were taken as the independent variables in each model.  **p*<.05 before Bonferroni correction, ***p<.*01 before Bonferroni correction, ****p<.*05 after Bonferroni correction (the p value cutoff for Bonferroni correction is p=0.05/8 tests=0.006). | | | | | | | | | |
